# An Ultra-Low-Cost RCL-Meter

**DOI:** 10.3390/s22062227

**Published:** 2022-03-14

**Authors:** Pedro M. C. Inácio, Rui Guerra, Peter Stallinga

**Affiliations:** 1 CEOT—Center for Electronics, Optoelectronics and Telecommunications, University of Algarve, Campus Gambelas, 8005-139 Faro, Portugal; pminacio@ualg.pt (P.M.C.I.); pjotr@ualg.pt (P.S.); 2 Department of Physics, University of Algarve, Campus Gambelas, 8005-139 Faro, Portugal; 3 Department of Electronics and Computer Engineering, University of Algarve, Campus Gambelas, 8005-139 Faro, Portugal

**Keywords:** impedance meter, RCL-bridges, portable instrument, AVR^®^ micro-controller, low-cost, internet of things

## Abstract

An ultra-low-cost RCL meter, aimed at IoT applications, was developed, and was used to measure electrical components based on standard techniques without the need of additional electronics beyond the AVR^®^ micro-controller hardware itself and high-level routines. The models and pseudo-routines required to measure admittance parameters are described, and a benchmark between the ATmega328P and ATmega32U4 AVR^®^ micro-controllers was performed to validate the resistance and capacitance measurements. Both ATmega328P and ATmega32U4 micro-controllers could measure isolated resistances from 0.5 Ω to 80 MΩ and capacitances from 100 fF to 4.7 mF. Inductance measurements are estimated at between 0.2 mH to 1.5 H. The accuracy and range of the measurements of series and parallel RC networks are demonstrated. The relative accuracy (*a*_r_) and relative precision (*p*_r_) of the measurements were quantified. For the resistance measurements, typically *a*_r_, *p*_r_ < 10% in the interval 100 Ω–100 MΩ. For the capacitance, measured in one of the modes (fast mode), *a*_r_ < 20% and *p*_r_ < 5% in the range 100 fF–10 nF, while for the other mode (transient mode), typically *a*_r_ < 20% in the range 10 nF–10 mF and *p*_r_ < 5% for 100 pF–10 mF. *a*_r_ falls below 5% in some sub-ranges. The combination of the two capacitance modes allows for measurements in the range 100 fF–10 mF (11 orders of magnitude) with *a*_r_ < 20%. Possible applications include the sensing of impedimetric sensor arrays targeted for wearable and in-body bioelectronics, smart agriculture, and smart cities, while complying with small form factor and low cost.

## 1. Introduction

The Internet of Things (IoT) entails a network of physical objects—‘things’—that are embedded with sensors, electronics, software, etc. for the purpose of communicating with other devices over the Internet. Caused by the sheer number of things connected in this way, these data-acquisition devices obviously need to be of low-cost and fulfill certain tasks: sensing, electronic processing and connecting to the Internet. Impedimetric sensor arrays are an emerging field of study that is concerned with sense, processing and casting the measured data to the Internet [1,2,3]. Typical applications are wearable and in-body electronics [4,5,6,7,8] and plants and smart agriculture [9,10,11,12,13]. A common aspect shared between different devices is that the electronic interfaces for detection, processing and connection to the Internet are mainly carried out by separate external systems specifically optimized for each of these functions. While designing a system with specific units may often be advantageous to enhance the overall performance of the device, it also leads to higher manufacturing cost. For example, most biosensors feature a transduction mechanism to couple the physical and/or chemical changes in the system under measurement to the electronic circuitry of the measuring device. The latter is specifically optimized for the sensing interface and is followed by an analog-to-digital conversion unit (ADC). In the most usual scenario, the end user has access to a plug-and-play instrument box. But even in this case, the transduction mechanism may require finding signal conditioning strategies in order to maximize the linear range of the analog signal and the corresponding measurement accuracy [14,15,16,17].

Furthermore, the measurement unit, which often replaces a benchtop instrument, must be designed for small form factor devices and a low power profile, while maintaining performance close to gold standard instruments. Application-specific integrated circuit (ASIC) based devices fulfill these requirements, such as the ones developed to perform online electrochemical impedance spectroscopy (EIS) for characterization of lithium-ion battery packs [18,19,20]. This technology can be extended to other applications in areas where size and power consumption are crucial. For instance, in our research we have been developing technology for using admittance spectroscopy to determine the physical state of plants [21]. However, this study relied on bulky and expensive lock-in detectors, not appropriate for an IoT implementation. A cheaper solution developed by S. Grassini is to use an Arduino-based electrochemical impedance spectroscopy (EIS) system [22] for in situ corrosion monitoring of metallic works of art [23]. The Arduino-based EIS is already a huge improvement over conventionally used RCL-bridges (resistance, capacitance, inductance) and lock-in detectors. Similarly, the ASIC-based miniaturized system for Online-EIS proposed by Manfredini [19] shows how versatile the ASIC device is, being capable of measuring not only the impedance of commercial batteries, but also capacitive and resistive sensors. The device is based on the SENSIPLUS, which is a System on a Chip (SoC) solution that uses minimal external hardware, and shows performance on a par with gold standard instruments. The Arduino has been also used as a platform to measure capacitances, as explained, for example, by Campbell [24]. It has been used to deploy a digital LCR meter [25] to measure single parameters (not combinations), although this depends on the known nominal values of external components.

The current report describes a very-low-cost solution for an admittance meter, resulting from an effort to lower the cost and the size of these instruments. Specifically, it uses a micro-controller for directly measuring admittance without the need for any additional electronic circuitry beyond the micro-controller hardware itself. The proposed implementation differs from the instruments developed by [22,24] in two main features: (i) it is based on a time-domain approach, since the measurements are performed with signal transients rather than with sinusoidal signals; (ii) it avoids the need for external circuits, since all the signal generation and detection are performed by the micro-controller. Moreover, when used in popular platforms such as Arduino, full functionality is available, from sensing to communicating, for a price that lies in the order of mere euros per unit, thus fulfilling the requirement of the Internet of Things. Additionally, it paves the way to cheaper wearable and in-body bioelectronic sensors, allowing a live feed of collected data from impedimetric sensor arrays to the IoT, constituting an all-in-one solution: Sensing, Processing and Connection to the Internet. The technique that we will describe here relies on determining the behavior after applying voltage steps, a standard technique used in circuit analysis, which can readily measure resistances and capacitances, either isolated or in series and parallel. It is based on the low-cost AVR^®^ micro-controller series commonly used in the Arduino^®^ platform. The accuracy and precision of the measurements are discussed based on the relative parameters of measurement uncertainty (*u*_r_), accuracy (*a*_r_) and precision (*p*_r_). All the C++ codes for Arduino and the MATLAB scripts used in this manuscript are available in the Supplementary Materials.

## 2. Materials and Methods

### 2.1. AVR^®^ Micro-Controllers Based RCL-Meter

**Figure 1:**
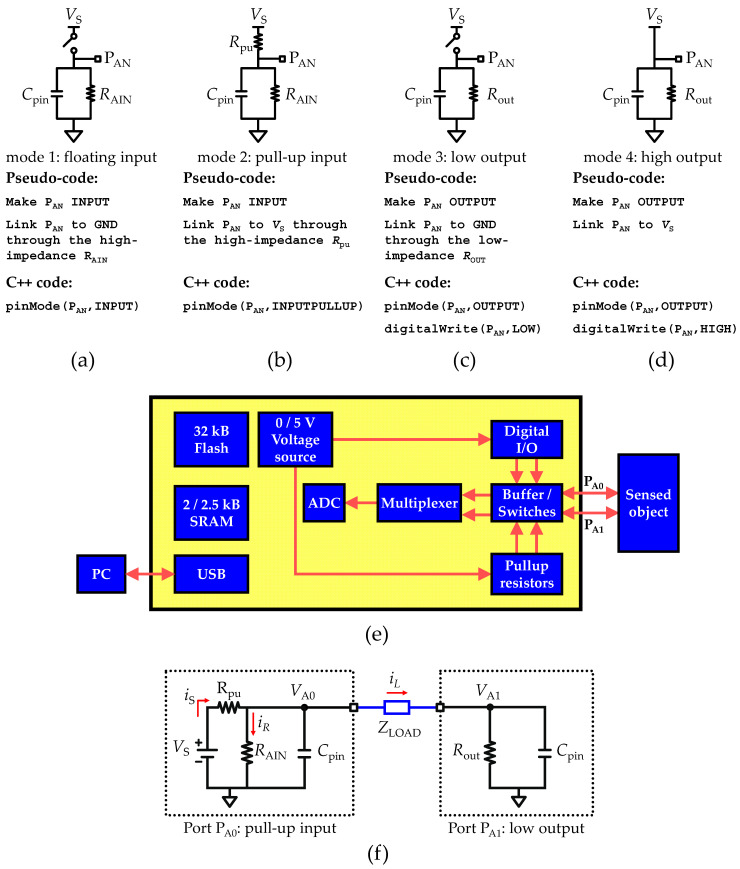
Equivalent circuits for the four operation modes available to configure each analog input/output (I/O) port. (**a**) mode 1, floating input. (**b**) mode 2, pull-up input. (**c**) mode 3, low output. (**d**) mode 4, high output. Each equivalent circuit is adapted from the schematics provided in the datasheets. (**e**) Block diagram of the proposed measurement system, including the serial communication and voltage source through USB interface to local PC, flash and SRAM memory, internal voltage source, and internal circuitry to program I/O ports to digital or alternate functionalities. (**f**) Equivalent circuit of two analog I/O ports bridged with a load impedance (*Z*_LOAD_).

Two development boards manufactured by Arduino^®^, namely the Uno and Leonardo boards, were used to develop the ultra-low-cost RCL-meter. The Arduino^®^ Uno board makes use of an ATmega328P micro-controller, and the Arduino^®^ Leonardo uses an ATmega32U4. Figure 1e shows the block diagram of the proposed measurement system based exclusively on the internal circuitry of the AVR^®^ micro-controller. Both ATmega328P [26] and ATmega32U4 [27] are low-power AVR^®^ 8-bit micro-controllers that share similar features, namely equal clock frequency up to 16 MHz, operational voltage range between 2.7 to 5.5 V, 32 kB of flash memory (enough to store the firmware and processing code), 2 kB (ATmega328P)/2.5 kB (ATmega32U4) of static random-access memory (SRAM) (to store the main variables and acquisition samples) and an analog-to-digital unit (ADC) with 10-bit resolution, the core of the system. Both the Uno and Leonardo boards were supplied through the local-PC connection via USB interface, and the default settings were used, meaning the reference voltage (*V*_REF_) is the internal voltage source of 5 V.

### 2.2. Analog I/O Operation Modes

The AVR^®^ micro-controller family provides access to several digital and analog ports with input and output (I/O) functionalities. Both digital and analog I/O ports share a common control unit design, often called “General Digital I/O” [26,27]. In this work, the digital I/O ports (PDN) are labeled by an alpha-numerical code, denoted by the letter “D” proceeded by the port bit number “N”, while the analog I/O ports (PAN) are represented by the letter “A”. The analog ports have exclusive access to the alternate functions, featuring an I/O source-measuring unit (SMU) and an analog-to-digital converter (ADC) unit. By default, *P*_AN_ ports can be configured into four distinct operation modes: (mode 1) floating input; (mode 2) pull-up input; (mode 3) low voltage output; (mode 4) high voltage output. Each operation mode shares the same basic electrical structure, consisting of the supply voltage source (*V*_S_), common ground (GND), a stray capacitance (*C*_pin_) and location of the I/O port (*P*_An_) for the SMU and ADC unit. By default, vs. is equal to the reference voltage (*V*_REF_). Figure 1 shows the equivalent circuit that describes each analog I/O port operation mode and was adapted from references [26,27]. Operation modes 1 and 2, shown in Figure 1a,b, respectively, share the same internal circuitry components to form a high-impedance input port due to the presence of the analog input resistance (*R*_AIN_). *C*_pin_ is also included in parallel with *R*_AIN_ to account for the stray capacitance. The unique difference between operation mode 1 and 2 is the state of the internal pull-up resistance (*R*_pu_), which is connected to an internal bias voltage. In the case of operating mode 1, the *R*_pu_ is inactive, leading to a floating input configuration, while in the case of operation mode 2, the *R*_pu_ sets a SMU configuration. This aspect will be further explored to implement the RCL-meter. Operation modes 3 and 4, shown in Figure 1c,d, relate to the “General Digital I/O” functions. Operation mode 3 consists of a floating and impedance low output configuration, and the operation mode 4 consists of a voltage source (*V*_S_), thus forcing a high output configuration. In the absence of load impedance connected to *P*_AN_, the configuration of the operation mode 4 sinks current through the parallel RC network formed by the output resistance (*R*_out_) and *C*_pin_. Table 1 shows the typical values of the internal components of the I/O ports [26,27].

The *R*_pu_ and *C*_pin_ values listed in Table 1 are representative values. In case of the *R*_pu_, both micro-controller datasheets [26,27] characterize the range of *R*_pu_ between 20 kΩ to 50 kΩ, and solely for the purpose of the ADC unit it refers a typical reference input resistance (*R*_REF_) value of 32 kΩ. As for the *C*_pin_, the micro-controller datasheets [26,27] do not provide a typical value; only a conceptual component is shown in the equivalent circuits of the analog input circuitry representing the overall stray capacitance (also named *C*_pin_). Therefore, the typical stray capacitance listed in Table 1 considers all capacitive sources in the internal analog input circuitry, such as: (i) the sampling and holder capacitance (*C*_S/H_), approximately equal to 14 pF and (ii) the input capacitance (*C*_i_) of each I/O pin, approximately equal to 10 pF. Both *C*_S/H_ and *C*_i_ are grounded; therefore, it is assumed that the parallel of both *C*_i_ ‖ *C*_S/H_ is the minimum value of *C*_pin_ and is approximately equal to *C*_pin_ ≅ *C*_i_ ‖ *C*_S/H_ ≅ 24 pF.

In practical terms, the operation mode of each port available on an AVR^®^ micro-controller must be configured according to the pseudo-code described in Figure 1 through a low-level instruction set, which is not a user-friendly environment. However, Arduino^®^ has developed an integrated development environment (IDE) platform with support of high-level C++ functions such as pinMode() and digitalWrite(), an easy and user-friendly method to configure the ports. Figure 1 describes the C++ code required to properly configure each operation mode. In the following sections, it will be shown how the four different configurations for the ports may be used to build an RCL bridge. First, it is shown how to measure isolated resistors, capacitors and inductors, and then how to measure combinations of resistors and capacitors in series or parallel.

### 2.3. Recording Circuit

The recording circuit to measure resistances, capacitances and inductances, either isolated or in series and parallel is composed of the same internal components by setting two I/O ports with alternate functions, namely *P*_A0_ and *P*_A1_ ports configured for pull-up input (mode 1) and low output (mode 3), respectively, and as described in Section 2.2. Figure 1f shows the equivalent circuit resulting from the combination of the internal circuitry of each I/O port bridged by a load impedance (*Z*_LOAD_). In the following Section 2.4, Section 2.5, Section 2.6, Section 2.7 and Section 2.8, the equivalent circuit shown in Figure 1f is detailed by replacing *Z*_LOAD_ with resistance, capacitance and inductance, either isolated or in series and parallel.

### 2.4. Measurements of an Isolated Load Resistance (R–Meter)

**Figure 2:**
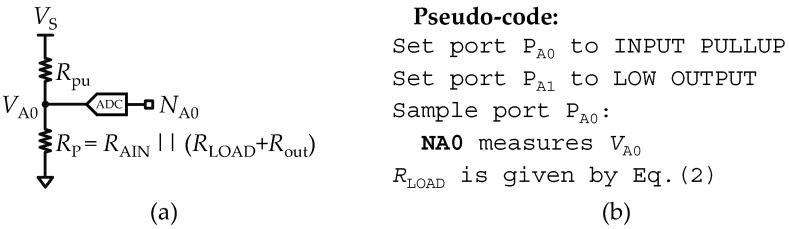
Set-up of a pure load resistance meter (R-meter). (**a**) Reduction of the equivalent circuit to a voltage divider circuit. (**b**) Pseudo-code used to implement the resistance meter mode.

Considering that in the equivalent circuit shown in Figure 1f, *Z*_LOAD_ is composed of an isolated load resistance (*R*_LOAD_), the pull-up resistor (*R*_pu_) at port *P*_A0_ drives a current source that will sink through the parallel resistance path (*R*_P_) formed by the analog input resistance (*R*_AIN_) and the sum of the load and output resistances (*R*_LOAD_ + *R*_out_). Thus, for circuit analysis purposes, in Figure 2a, a reduction of the entire equivalent circuit to a voltage divider circuit is shown. In Figure 2a, the variable *N*_A0_ is introduced as the digital counterpart of the analog variable *V*_A0,_ where *N*_A0_ = ⌊*N*_max_·(*V*_A0_/*V*_S_)⌋. The stray capacitances (*C*_pin_) were neglected from the equivalent circuit, since on DC measurements, the capacitances behave as open circuit. In addition, to avoid interference of any stray capacitance, the procedure is to implement a reasonable delay time (say 1 ms) after setting the port *P*_A0_ to operation mode 2 (pullup input). This procedure assures that the stray capacitances are open circuit (*t* ≫ *τ* = *R*_T_*C*_pin_). Using *C*_pin_ = 25 pF and *R*_pu_ = 32 kΩ, one obtains *τ* < 1 µs.

In Figure 2a, *V*_A0_ is the measured voltage at port *P*_A0_ and is given by:

(1) $$V_{A0}=V_{S}\frac{R_{p}}{R_{\mathrm{pu}}+R_{p}}$$

where *R*_P_ is given by *R*_AIN_ || (*R*_LOAD_ *+ R*_out_), and vs. is equal to the reference voltage source (*V*_REF_). Solving the voltage divider Equation (1), *R*_LOAD_ is found as:

(2) $$R_{\mathrm{LOAD}}=\frac{R_{\mathrm{AIN}}R_{\mathrm{out}}-K\left(R_{\mathrm{AIN}}+R_{\mathrm{out}}\right)}{K-R_{\mathrm{AIN}}},K=R_{\mathrm{pu}}\frac{V_{A0}}{V_{S}-V_{A0}}$$

where *K* is an auxiliary variable used to shorten the length of Equation (2). All the parameters described in Equation (2) are known values, and are available on the datasheet of the micro-controller. The pseudo-code for a routine to measure *R*_LOAD_ is described in Figure 2b.

### 2.5. Measurements of an Isolated Load Capacitance (C–Meter)

Two methodologies are reported in this section: (i) fast acquisition mode, which is based on the immediate response of the *C*_LOAD_ charging cycle, and (ii) transient acquisition mode, based on measuring the charging time until a threshold voltage is reached. It is essential that prior to initiating measurements of *C*_LOAD_, all capacitive components are fully discharged. A typical procedure to discharge all capacitances is achieved by configuring the operation mode of the two I/O ports in use to low output (mode 3) and waiting, for example, a period of Δ*t* ≥ 1 ms.

#### 2.5.1. Fast Acquisition Mode of an Isolated Load Capacitance

**Figure 3:**
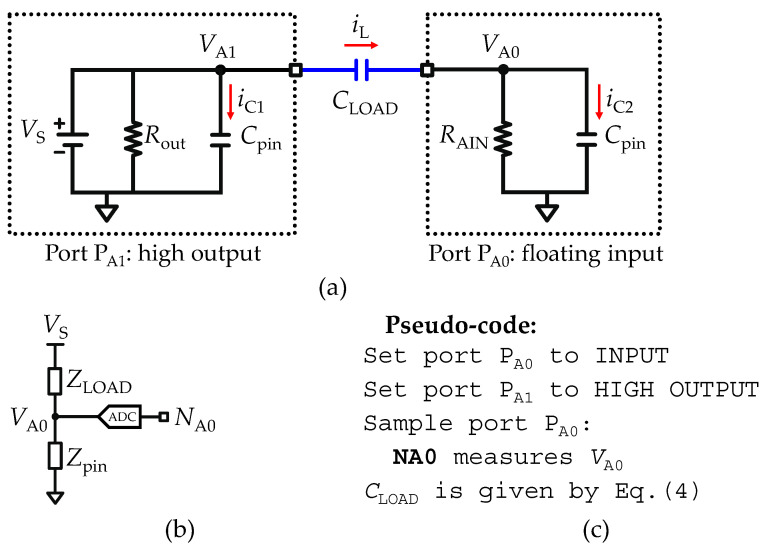
Set-up of a pure load capacitance meter (*C*-meter). (**a**) Equivalent circuit of two analog I/O ports bridged with a load capacitance (*C*_LOAD_). (**b**) Reduction of the equivalent circuit to an impedance divider. (**c**) Pseudo-code used to implement the fast acquisition mode.

As an exception to all other methods presented in this manuscript, the fast acquisition mode to measure an isolated load capacitance (*C*_LOAD_) uses a different internal circuitry. Therefore, Figure 3a shows the equivalent circuit resulting from the combination of the internal circuitry of two I/O ports, *P*_A0_ and *P*_A1_, bridged by a pure load capacitance (*C*_LOAD_). Essentially, the port *P*_A1_ is set in high output (mode 4) and links the voltage source (*V*_S_) to the *C*_LOAD_ terminal behaving as an input current source (*i*_S_), and the port *P*_A0_ is set to floating input (mode 1), consisting of a measuring unit that links the input current to the ground through a high impedance resistor (*R*_AIN_). Both the *P*_A1_ and *P*_A0_ ports consider the leakage current path to the ground through a stray capacitance (*C*_pin_). However, the *C*_pin_ located at *P*_A1_ can be neglected, since the characteristic time constant (*τ*_A1_) of *P*_A1_ is *τ*_A1_ = *R*_out_*C*_pin_ ≅ 15 ns, and therefore, because the fastest clock cycle (*τ*_clk_) of the micro-controllers in use are approximately equal to *τ*_clk_ *≈* 5*τ*_A1_, after a single clock cycle the voltage in port *P*_A1_ (*V*_A1_) is stationary.

For circuit analysis purposes, assuming all capacitances are fully discharged at the instant *t* = 0, all capacitances are shorted and all currents flow through the capacitive path. In these circumstances, the circuit analysis is simpler considering that at *t* = 0 the current path flow exclusively through the capacitive path (*C*_T_) formed by the load (*C*_LOAD_) and stray (*C*_pin_) capacitors in series, given by *C*_T_ = *C*_LOAD_*C*_pin_/(*C*_LOAD_ + *C*_pin_). This allows the reduction of the entire equivalent circuit to a voltage divider, as shown in Figure 3b, and is only valid for measuring the impulse response. Thus, *V*_A0_ must be read immediately after defining *P*_A0_ to floating input and *P*_A1_ to high output.

One approach to find the load capacitance is through the analysis of the charge level (*Q*) of the circuit. Since both *C*_LOAD_ and *C*_pin_ are in series, the charge level must be equal in both capacitances (*Q*_LOAD_ = *Q*_pin_), thus allowing the expression of *V*_A0_ as:

(3) $$V_{A0}=V_{S}\frac{C_{\mathrm{pin}}}{C_{\mathrm{pin}}+C_{\mathrm{LOAD}}}$$

where the solution given by Equation (3) assumes *Q*_LOAD_ = *C*_LOAD_ (*V*_S_ − *V*_A0_) and *Q*_pin_ = *C*_pin_*V*_A0_. Rearranging (3), *C*_LOAD_ is found as:

(4) $$C_{\mathrm{LOAD}}=C_{\mathrm{pin}}\frac{V_{A0}}{V_{S}-V_{A0}}$$

The size of *C*_pin_ is made available through the datasheet of the micro-controller, and is typically 25 pF, while vs. is equal to the reference voltage source (*V*_REF_), and *V*_A0_ is the measured voltage at port *P*_A0_. Thus, all parameters are known, and *C*_LOAD_ can be determined. Lastly, the fast acquisition mode methodology is limited by the size of the *C*_pin_, such that, if *V*_A0_ → *N*_max_, the maximum range of (4) is an asymptote with *C*_LOAD_ → ∞. Therefore, an approximation to determine the range of measurable *C*_LOAD_ is:

(5) $$\frac{C_{\mathrm{pin}}}{N_{\max}-1}\leq C_{\mathrm{LOAD}}\leq C_{\mathrm{pin}}\left(N_{\max}-1\right)$$

where the total range is obtained for 1 ≤ *N*_A0_ < *N*_max_ − 1. The pseudo-code for a routine to measure *C*_LOAD_ through the fast acquisition mode is described in Figure 3c.

#### 2.5.2. Transient Acquisition Mode of an Isolated Load Capacitance

As pointed out before, the fast-acquisition mode methodology has limitations for large capacitances, thus requiring a different measurement strategy, namely monitoring the transient during the charging cycle of a *C*_LOAD_ bridging the ports *P*_A0_ and *P*_A1_ in the equivalent circuit shown in Figure 1f. The stray capacitances (*C*_pin_) are not larger than some tens of pico-Farads (pF) and can therefore be neglected.

**Figure 4:**
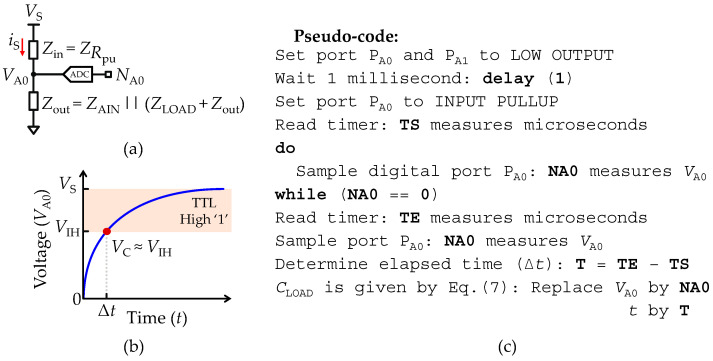
Set-up for recording a pure load capacitance (*C*_LOAD_) through the transient acquisition mode. (**a**) Impedance representation of the reduced equivalent circuit. (**b**) Illustration of the step response in voltage measured at the input terminal of *C*_LOAD_. *V*_IH_ is defined in Table 1. (**c**) Pseudo-code used to implement the transient acquisition mode.

The remaining circuit analysis is simpler and is preferably done using the impedance analysis. Figure 4a shows the impedance representation of the equivalent circuit, where *Z*_in_ represents the impedance due to the pull-up resistance (*R*_pu_) at the input port *P*_A0_, and *Z*_out_ is the output impedance formed by the parallel RC network between the analog input resistance (*R*_AIN_) and the series RC network (*Z*_LOAD_ + *R*_out_). *Z*_LOAD_ is the impedance representation of the load capacitance (*C*_LOAD_). Therefore, an estimation of *C*_LOAD_ is obtained using the step response of the impedance divider circuit shown in Figure 4b, which is given by:

(6) VA0(t)=VS(1−e−tτ), τ =RTCLOAD

where *R*_T_ is the total resistance path contributing for the potential difference on the capacitance terminals given by *R*_T_ = *R*_AIN_ || *R*_pu_ *+ R*_out_. Thus, *C*_LOAD_ can be found at any time *t* by rearranging Equation (6) to:

(7) $$C_{\mathrm{LOAD}}=\frac{t}{R_{T}\cdot \ln\left(\frac{V_{S}}{V_{S}-V_{A0}\left(t\right)}\right)}$$

where *t* is the time since starting the charging of the capacitance. All parameters in Equation (7) are known except for the time (*t*). Thus, to find the *C*_LOAD_ value, the elapsed time (Δ*t*) must be determined, since *t* = 0 until a threshold voltage (*V*_th_) is reached. For instance, solving Equation (6) with *t* = *τ* allows the definition of *V*_th_ = (1 *− e^−^*^1^)*V*_S_ and therefore solves Equation (7). The elegant procedure for testing *V*_th_ makes use of the built-in transistor-transistor logic (TTL) unit to monitor *V*_A0_ by setting the TTL unit high voltage threshold (*V*_IH_) to the *V*_th_ (*V*_th_ = *V*_IH_), allowing not only the avoidance of the computation of the *V*_th_, but also the enhancement of the accuracy of the measurement. In practical terms, the elapsed time (Δ*t*) since *C*_LOAD_ initiates the charging cycle (*t* = 0) until the TTL unit changes to the logical state high ‘1’ (*V*_A1_ ≥ *V*_IH_) must be measured, as well as proceeding to the reading of *V*_A0_ using the ADC unit (*N*_A0_). Then, *C*_LOAD_ is found by replacing *V*_A0_ and *t* by the measured values of *N*_A0_ and Δ*t* in Equation (7). Figure 4c depicts the voltage step response of the impedance divider circuit. The highlighted region delimited between the voltage level *V*_IH_ and vs. represents the operation region where the TTL logic unit changes of logic state low ‘0’ to high ‘1’. For reference, Table 1 includes the typical values of *V*_IH_ for the ATmega328P and ATmega32U4 micro-controllers.

Additionally, the transient acquisition mode methodology is limited by the characteristic time constant (*τ*) of the circuit, such that if *τ* → 0, the charging velocity (d*v*/d*t*) of the *C*_LOAD_ increases to proportions where high temporal resolution is required to measure the *N*_A0_ accurately, since *N*_A0_ → *N*_max_ just before the first reading is taken, causing the ADC unit to overflow. An approximation of the ranging limits of the transient acquisition mode is given by:

(8) $$\frac{\Delta t}{R_{T}\cdot \ln\left(N_{\max}\right)}\leq C_{\mathrm{LOAD}}\left(t\right)\leq \frac{\Delta t}{R_{T}\cdot \ln\left(\frac{N_{\max}}{N_{\max}-1}\right)}$$

where in Equation (8) the lower and upper resolution of the ADC unit (*N*_A0_) are solved assuming 1 ≤ *N*_A0_ ≤ *N*_max_ − 1. Using the typical values provided in Table 1, the detection limit of the interface assuming the fastest reading of the ADC unit could be achieved after one clock-cycle (Δ*t* = 62.5 ns). Then, the minimum range of the transient acquisition method varies between 214 fF ≤ *C*_min_ ≤ 392 fF with 22.4 kΩ ≤ *R*_pu_ ≤ 41.6 kΩ. Nevertheless, the impact of the stray capacitances (*C*_pin_) was neglected, and therefore such a range is merely indicative, since *C*_pin_ ≫ *C*_min_. As for the maximum range of the transient acquisition method, the maximum Δ*t* is limited by the size of the unsigned long variables given by Δ*t* = (2^32^ − 1) × 1 µs ≅ 4295 s. Thus, the maximum range corresponds to a battery-like storage unit rather than a capacitor, as it is some hundreds of farads. The pseudo-code of a routine to perform the measurement of *C*_LOAD_ through the transient acquisition mode is described in Figure 4c.

### 2.6. Measurements of a Serial RC Network (RC–Meter Mode)

Considering that in the equivalent circuit shown in Figure 1f, *Z*_LOAD_ is composed by a series RC network, the strategy to extract *C*_LOAD_ and *R*_LOAD_ resembles the previously described techniques to measure a pure resistor in Section 2.4 and a pure capacitor through the transient acquisition mode in Section 2.5.2. However, the TTL-based technique used to determine *C*_LOAD_ cannot guarantee that a TTL transition will occur in the transient response of the circuit, since the voltage *V*_A0_ might start at a value above the TTL threshold (*V*_IH_). Instead, the monitoring process of the transient response must be carried out exclusively using the analog functions available on the AVR^®^ micro-controller ports. First, the *C*_LOAD_ must be completely discharged, which requires defining both ports *P*_A0_ and *P*_A1_ to low output configuration for a reasonable time, and then defining *P*_A0_ to a high-impedance SMU, while maintaining *P*_A1_ as low output. For the sake of simplicity, the stray capacitances (*C*_pin_) are neglected, which is allowed if they are not larger than some tens of pico-farads (pF).

**Figure 5:**
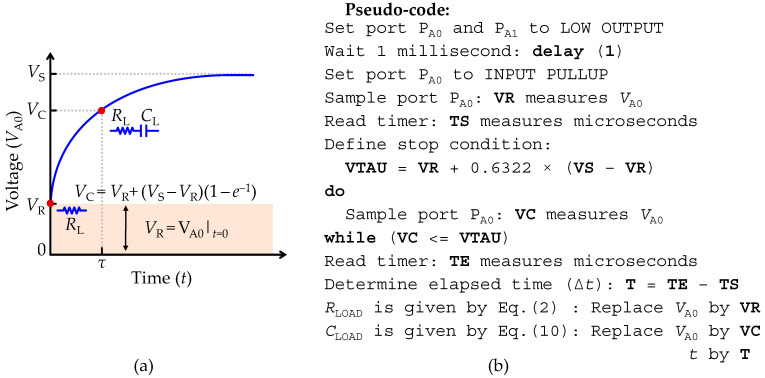
Set-up for recording a load impedance (*Z*_LOAD_) formed by a serial RC network. (**a**) Illustration of the transient response to a voltage step at the input terminal (*P*_A0_). (**b**) Pseudo-code used to implement the measurement of a serial RC network.

With *C*_LOAD_ fully discharged, after setting the port *P*_A0_ to a high-impedance SMU (*t* = 0), a voltage step is applied on the serial RC circuit. Since *C*_LOAD_ is empty, it behaves as a short-circuit, which results in an equivalent circuit, as shown in Figure 2a, thus allowing the extraction of the *R*_LOAD_ value through use of the technique described in Section 2.4 to measure a pure resistor (note that the port configuration is the same). The measured voltage (*V*_A0_) at the instant *t* = 0 defines an offset voltage (*V*_R_). For any instant *t >* 0, *C*_LOAD_ starts to accumulate charge, and the same circuit analysis described in Section 2.5.2 applies to determine the transient dynamics of the voltage step response. For *t* → ∞, *C*_LOAD_ is fully charged and behaves as an open circuit, thus forcing the current to flow exclusively through the analog input resistance (*R*_AIN_), as represented in Figure 1f by the current path ‘*i*_R_’. Thus, considering the influence of the charging current (*i*_L_) due to the series *R*_LOAD_, an estimation of *C*_LOAD_ is obtained using the step response of the equivalent circuit shown in Figure 1f that is given by:

(9) VA0(t)=VR+VREF(1−e−tτ), VR=VSRpu(Rout+RLOAD)(RAIN ‖ Rpu)Rout+RLOAD+RAIN ‖ Rpu,

where *V*_R_ is the offset voltage due to the series *R*_LOAD_, *V*_REF_ is the new reference voltage of the circuit given by *V*_REF_ = vs. − *V*_R_, *τ* is the characteristic time constant of the equivalent circuit given by the *τ* = *R*_T_*C*_LOAD_ and *R*_T_ is the total resistance path contributing to the potential difference on the capacitance terminals given by *R*_T_ = *R*_AIN_ || *R*_pu_ *+ R*_LOAD_ *+ R*_out_. Thus, *C*_LOAD_ can be found at any time *t* by rearranging Equation (9) to:

(10) $$C_{\mathrm{LOAD}}=\frac{t}{R_{T}\ln\left(\frac{V_{\mathrm{REF}}}{V_{\mathrm{REF}}-V_{A0}\left(t\right)}\right)}$$

where *t* is the time that takes charges to accumulate in the capacitance. Figure 5a illustrates the transient response to a voltage step expressed by Equation (10), where the highlighted region consists of the offset voltage (*V*_R_) due to the bridge of ports *P*_A0_ and *P*_A1_ with the serial RC network. As for the reading capacitance voltage level (*V*_C_) it is determined when *t* = *τ* = *R*_T_*C*_LOAD_, and is given by *V*_C_ = *V*_R_ + (1 − *e^−^*^1^)(*V*_S_ − *V*_R_). Therefore, all parameters in Equation (10) are known except for the time (*t*). To extract *C*_LOAD_, a routine to determine the elapsed time (Δ*t*) until the measured voltage (*V*_A1_) is greater than or equal to *V*_C_ (*V*_A0_ ≥ *V*_C_) must be implemented. Figure 5b depicts the pseudo-code of a routine to implement the measurement of a serial RC network. In practical terms, *C*_LOAD_ is found by replacing in Equation (10) *V*_R_ with the measured value of *N*_A0_ at *t* = 0 (*N*_R_), vs. with the maximum resolution of the ADC unit (*N*_max_) and *V*_A0_ and *t* with the measured value of *N*_A0_ and Δ*t* after *V*_A0_ ≥ *V*_C_, respectively.

### 2.7. Measurements of a Parallel RC Network (RC–Meter Mode)

**Figure 6:**
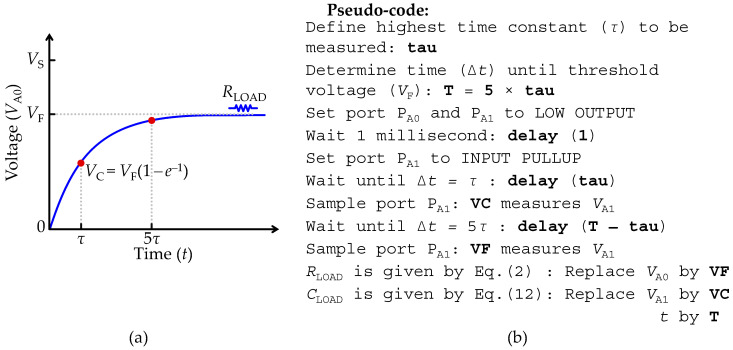
Set-up for recording a load impedance (*Z*_LOAD_) formed by a parallel RC network. (**a**) Illustration of the transient response to a voltage step at the input terminal (*P*_A0_). (**b**) Pseudo-code used to implement the measurement of a parallel RC network.

Considering that in the equivalent circuit shown in Figure 1f, *Z*_LOAD_ is composed of a parallel RC network (*C*_LOAD_║*R*_LOAD_), the strategy to extract *C*_LOAD_ and *R*_LOAD_ resembles the previously described technique in Section 2.6, although it must be noted that the change of the load capacitance (*C*_LOAD_) from serial mode to parallel mode causes *R*_LOAD_ to saturate at the reference voltage (*V*_F_ = *V*_R_) level when *t* → ∞ and the voltage offset is null at *t* = 0. Thus, the step response of the equivalent circuit shown in Figure 1f is given by:

(11) VA0(t)=VF(1−e−tτ),

where *V*_F_ is given by the expression of *V*_R_ that is defined in Equation (9), *τ* is the characteristic time constant of the equivalent circuit given by *τ* = *R*_T_*C*_LOAD_ and *R*_T_ is the total resistance path given by *R*_T_ = (*R*_AIN_ || *R*_pu_) || (*R*_LOAD_ + *R*_out_). Figure 6a illustrates the transient response to a voltage step expressed by Equation (11). For *t* → ∞, *C*_LOAD_ is fully charged and behaves as an open circuit, forcing the current (*i*_L_) represented in the equivalent circuit shown in Figure 1f to flow exclusively through the *R*_LOAD_ to the ground. Therefore, the extraction of *R*_LOAD_ is analytically indeterminate, unless an approximation is made, such as, considering for any period (Δ*t*) larger than five times the characteristic time constant (*τ*) (Δ*t* > 5*τ*), Equation (11) is approximately equal to *V*_A0_ = *V*_F_(1 − *e*^−5^) ≈ *V*_F_, where the term (1 − *e*^−5^) ≈ 0.993 shows that the approximation has a maximum error of 0.7% when determining the *R*_LOAD_ value. Thereby, assuming for any Δ*t* > 5*τ* the plateau *V*_A0_ = *V*_F_ is reached, the *R*_LOAD_ value is obtained using the technique described in Section 2.4 to measure a pure resistor, replacing in Equation (2) *V*_A0_ by *V*_F_, where *V*_F_ is the measured voltage for any instant Δ*t* > 5*τ*. The next step is to determine the *C*_LOAD_ value through the arrangement of Equation (11) with *τ* = *R*_T_*C*_LOAD_ by:

(12) $$C_{\mathrm{LOAD}}=\frac{t}{R_{T}\cdot \ln\left(\frac{V_{F}}{V_{F}-V_{A0}\left(t\right)}\right)}$$

where *t* is any instant of the step-response and *V*_A0_ the correspondent measured voltage. The determination of *V*_F_ is critical to find both *C*_LOAD_ and *R*_LOAD_ values. The simplest approach to find *V*_F_ using the transient response to a voltage step is to define the maximum characteristic time constant (*τ*_max_) to measure. In these circumstances all parameters are known, and both *C*_LOAD_ and *R*_LOAD_ values can be measured using Equations (2) and (10). Figure 6b depicts the pseudo-code of a routine to implement the measurement of a parallel RC network.

### 2.8. Measurements of an Isolated Load Inductance (L–Meter Mode)

**Figure 7:**
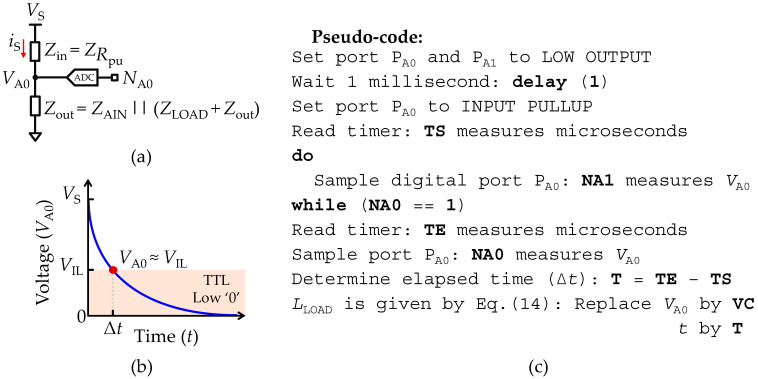
Set-up for recording an isolated load inductance (*L*_LOAD_) through the transient acquisition mode. (**a**) Impedance representation of the reduced equivalent circuit. (**b**) Illustration of the step response in voltage measured at the input terminal of *L*_LOAD_. *V*_IL_ is defined in Table 1. (**c**) Pseudo-code used to implement the inductance transient acquisition mode.

Considering that in the equivalent circuit shown in Figure 1f, *Z*_LOAD_ is composed of an isolated load inductance (*L*_LOAD_), and that for the sake of simplicity, the stray capacitances (*C*_pin_) are neglected, which is allowed if they are not larger than some tens of pico-farads (pF), the circuit analysis is simpler than and preferable to using the impedance analysis. Figure 7a shows the impedance representation of the equivalent circuit, where *Z*_in_ represents the impedance due to the pull-up resistance (*R*_pu_) at the input port *P*_A1_, and *Z*_out_ is the output impedance formed by the parallel RC network between the analog input resistance (*R*_AIN_) and the series RC network (*Z*_LOAD_ + *R*_out_). *Z*_LOAD_ is the impedance representation of the load inductance (*L*_LOAD_), given by *Z*_LOAD_ = *jωL*_LOAD_. Therefore, an estimation of *L*_LOAD_ is obtained using the step response of the impedance divider circuit shown in Figure 7a, which is given by:

(13) VA0(t)=VS(1−e−tτ), τ =LLOAD/RT,

where *R*_T_ is the total resistance path contributing to the potential difference on the inductance terminals given by *R*_T_ = *R*_AIN_ || *R*_pu_ *+ R*_out_. Thus, *L*_LOAD_ can be found at any time *t* by rearranging Equation (13) to:

(14) $$L_{\mathrm{LOAD}}=\frac{{tR}_{T}}{\ln\left(\frac{V_{S}}{V_{S}-V_{A0}\left(t\right)}\right)},$$

where *t* is the time that takes charges to accumulate in the inductance. Except for the time (*t*), all parameters in Equation (14) are known and available in Table 1. Therefore, the strategy to solve the time (*t*) of Equation (14) requires a similar approach, as previously described for the measurement of an isolated capacitance through the transient response. In this case, it must be monitored when the threshold voltage (*V*_th_) over *L*_LOAD_ is approximately equal to *V*_th_ = *V*_S_·(1 − *e*^−1^). As for the isolated capacitance measurement described in Section 2.5.2, the elegant and simpler procedure for testing *V*_th_, involves making use of the built-in transistor-transistor logic (TTL) unit to monitor *V*_A0_ by setting the TTL unit low voltage threshold (*V*_IL_) the *V*_th_ (*V*_th_ = *V*_IL_). In practical terms, the elapsed time (Δ*t*) since *L*_LOAD_ initiates the cycle (*t* = 0) until the TTL unit changes to the logical state low ‘0’ (*V*_A0_ ≤ *V*_IL_) must be measured, proceeding afterwards to the reading of *V*_A0_ using the ADC unit (*N*_A0_). Then, *L*_LOAD_ is found by replacing *V*_A0_ and *t* with the measured values of *N*_A0_ and Δ*t* in Equation (14). Figure 7b depicts the voltage step response of the impedance divider circuit. The highlighted region delimited between the voltage level *V*_IL_ and vs. represents the operation region where the TTL logic unit changes of logic state high ‘1’ to low ‘0’.

The measurement range of the inductance is limited due to the ADC unit resolution, where the technique described above to monitor the transient response to a voltage step is limited by the characteristic time constant (*τ*) of the circuit, such that if *τ* → 0, the charging current velocity (d*i*/d*t*) of the *L*_LOAD_ decreases to such proportions that accurately measuring the *N*_A0_ requires high temporal resolution, since *N*_A0_ → 0 just before the first reading is taken, causing the ADC unit to overflow. An approximation of the ranging limits of the transient acquisition mode is given by:

(15) $$\frac{\Delta t\cdot R_{T}}{\ln\left(N_{\max}\right)}\leq L_{\mathrm{LOAD}}\left(t\right)\leq \frac{\Delta t\cdot R_{T}}{\ln\left(\frac{N_{\max}}{N_{\max}-1}\right)}$$

where in Equation (15) the lower and upper resolution of the ADC unit (*N*_A0_) are solved assuming 1 ≤ *N*_A0_ ≤ *N*_max_ − 1. With the same values and assumptions used for the transient C-method, the range of measurement will vary between 0.2 mH ≤ *L*_LOAD_ ≤ 1.5 H with *R*_pu_ = 22.4 kΩ, and 0.4 mH ≤ *L*_LOAD_ ≤ 2.7 H with *R*_pu_ = 41.6 kΩ. These measurable values of *L*_LOAD_ are not very useful in real life applications, and for that reason in this manuscript only the methodology is provided as proof of concept to perform measurements with AVR^®^ micro-controllers of an isolated inductance. In spite of this, the pseudo-code is provided in Figure 7c to implement the measurements of an isolated *L*_LOAD_ through the transient acquisition mode built-in AVR^®^ micro-controllers.

### 2.9. Data Acquisition and Analysis

The open-source Arduino^®^ IDE software was used to program and upload the scripts on the AVR^®^ micro-controllers. All routines that were programmed to perform the measurements of the RCL-meter are based on the pseudo-codes previously described in each method. The data acquisition was carried out with the serial interface made available on the Arduino^®^ IDE software. The collected data were handled with MATLAB^®^ to perform the data analysis. All measurements were performed at room temperature, about 25 °C.

### 2.10. Noise and Uncertainty of the Measurements

To achieve the highest measurement accuracy of the RCL parameters, the parasitic capacitances must be minimized by leaving a space of two analog pins between the measured ports, e.g., ports *P*_A0_ and *P*_A3_, and shorten the connection cables to a minimum size.

Additionally, the impact of noise sources in the measurements must be considered. This includes the thermal and 1/*f* contributions, which are not easily modelled, but are included in the global noise measurements. The measured noise of the voltage source (Δ*V*_n_) was 6.7 mV (with the Arduino board powered through the USB interface connected to a local-PC), comparable to the digitalization uncertainty of the ADC (5 mV) described below. This value permits an enhancement of the accuracy of the ADC unit by oversampling techniques. This shows that the digitalization dominates over thermal and 1/*f* noise contributions. Thus, in this analysis we only calculate the effect of digitalization uncertainty. Using the default ADC sampling time of 100 µs, the digitalization uncertainty of a 10-bit ADC unit relative to the reference voltage (*V*_REF_) of 5 V, the least significant bit (LSB) voltage is 4.9 mV. To analyze the impact of this digitalization uncertainty, the relative uncertainty (*u*_r_) associated with the analog to digital round off was determined as:

(16) $$u_{r}=\frac{f(N_{\mathrm{meas}}+0.5)-f(N_{\mathrm{meas}}-0.5)}{f(N_{\mathrm{meas}})},$$

where *f*(*N*) represents one of the previous Equations (2), (4), (7), (10), (12) and (14) used to determine the *R*_LOAD_, *C*_LOAD_ and *L*_LOAD_ values. Then, the oversampling technique allow for reducing the digitalization uncertainty (*u*_r_) by a factor of 1/*N* but limited by other sources of uncertainty (noise).

### 2.11. Relative Accuracy and Precision of the Measurements

The errors associated with the accuracy and precision of the measurements were analyzed through the relative accuracy (*a*_r_) and the relative precision (*p*_r_). The relative precision (*p*_r_) measures the dispersion of the measured impedance values (*Z*_meas_) normalized to their average ($\bar{Z}$_meas_) and was estimated by *p*_r_ = SD(*Z*_meas_)/$\bar{Z}$_meas_, where SD represents the standard deviation of the measured values. The relative accuracy (*a*_r_) measures the closeness of the measured *Z*_meas_ to the true or reference value, *Z*_nominal_, and was estimated by *a*_r_ = |$\bar{Z}$_meas_ − *Z*_nominal_|/*Z*_nominal._

### 2.12. Linearization of the ADC Unit

**Figure 8:**
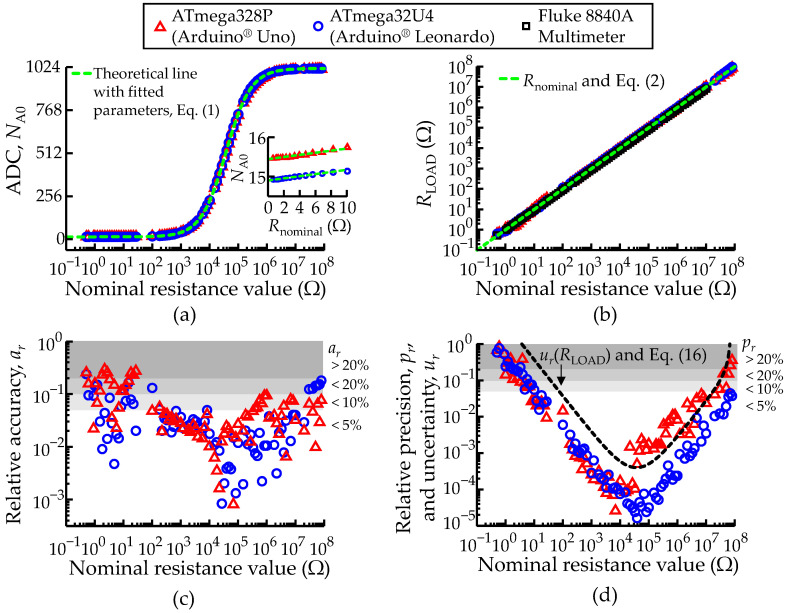
Comparison between the ATmega328P and ATmega32U4 AVR^®^ micro-controllers configured to record an isolated load resistance (*R*_LOAD_). (**a**) Measured ADC unit discrete values at port *P*_A0_ (*N*_A0_). Each sample consists of an average of 100 consecutive measurements. (**b**) Measured load resistance (*R*_LOAD_) values according to Equation (2). The green dashed lines represent the theoretical lines. (**c**) Relative accuracy (*a*_r_) and (**d**) relative precision (*p*_r_) of the measurements in function of *R*_nominal_. The black dashed line represents the relative uncertainty (*u*_r_) of the *R*_LOAD_ measurements according to Equation (16). The white and grey shading areas highlight the levels of *u*_r_, *a*_r_ and *p*_r_ better than 5%, 10% and 20%. A legend to describe the color scheme used in all plots of Figure 8 was included.

Work was done to improve the linearization of the ADC unit output. By linearization is here meant the maximization of the correlation between measured and nominal values, which depends on the correct knowledge of the micro-controller’s parameters. Since all methods presented previously to measure an unknown load impedance (*Z*_LOAD_) use the same ports configuration (except for the measurements of an isolated load capacitance using the fast acquisition mode), the simplest method to optimize the linearization of the measurements is to replace *Z*_LOAD_ with a pure known load resistance (*R*_LOAD_) and use different resistor sizes to test and maximize the range and accuracy of the ADC unit. Then, Equation (2) must be used as a fitting function, where the *R*_LOAD_ values must be replaced by the nominal values provided by the manufacturer of the resistor, and *V*_A0_ and vs. by the ADC unit values. Figure 8a shows the *N*_A0_ values collected with through-hole resistors bridging two ports of the micro-controllers. All samples consist of an average of 100 consecutive measurements. The data were distributed in a logarithmic scale along the horizontal axis according to the nominal resistance value provided by the manufacturer. The red triangles represent the samples measured with the ATmega328P and the blue circles represent the ATmega32U4. Ordinary Least Squares (OLS) were used to fit the data shown in Figure 8a, but with a small twist, which consisted of applying the natural logarithm (ln) to the OLS objective fit function and to the measured *N*_A0_ values. This proved able to cope better with the large range of resistance values, encompassing several orders of magnitude. Then, to extract the optimized values of *R*_AIN_, *R*_out_ and *R*_pu_, the user must provide a guess estimation and use a nonlinear programming solver to find the minimum values of the OLS objective function around the estimated values, taken from the manufacturer datasheets. Table 2 shows the extracted values of *R*_AIN_, *R*_out_ and *R*_pu_ for both ATmega328P and ATmega32U4 AVR^®^ micro-controllers used in the current work.

Figure 8a shows the ADC readings as a function of the nominal ADC values. Actual values for both ATmega328P and ATmega32U4 micro-controllers are represented by symbols, and the corresponding fitted lines from Equation (2) (overlapped) in dashed green. The figure inset provides a closer view of the measured samples between 0.5 Ω to 10 Ω. The fitted parameters allow a broad working range of about 8 orders of magnitude for the measured resistance. Otherwise, using typical values provided by the manufacturer would result in a working range of only 2 orders of magnitude. This procedure was performed only once and the values obtained for *R*_AIN_, *R*_out_ and *R*_pu_ were used in all the subsequent measurements.

**Figure 9:**
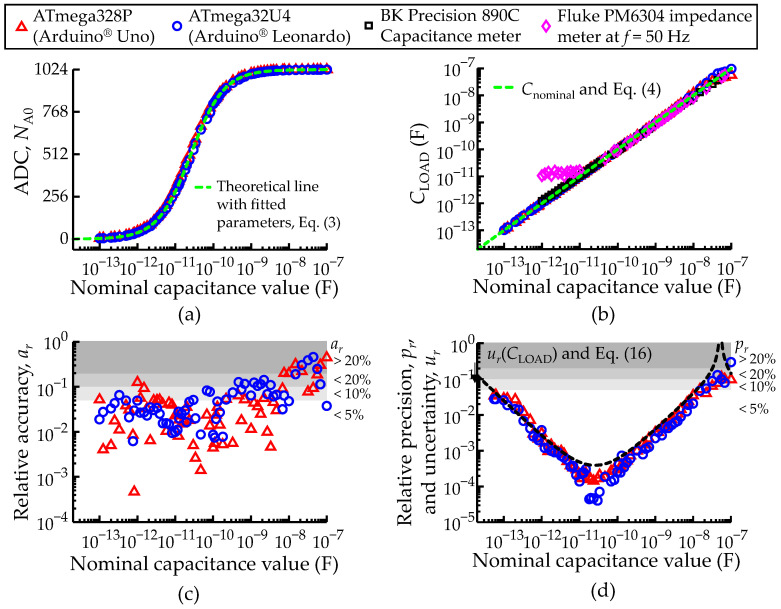
Comparison between the ATmega328P and ATmega32U4 AVR^®^ micro-controllers configured to record an isolated load capacitance (*C*_LOAD_) through the fast acquisition mode. (**a**) Measured ADC values at port *P*_A0_ (*N*_A0_). Each ADC sample consists of an average of 100 consecutive measurements. (**b**) Measured load capacitance (*C*_LOAD_) given by Equation (4). The green dashed lines represent the theoretical lines. (**c**) Relative accuracy (*a*_r_) and (**d**) relative precision (*p*_r_) of the measurements in function of *C*_nominal_. The black dashed line represents the relative uncertainty (*u*_r_) of the *C*_LOAD_ measurements according to Equation (16). The white and grey shading areas highlight the levels of *u*_r_, *a*_r_ and *p*_r_ better than 5%, 10% and 20%. A legend to describe the color scheme used in all plots of Figure 9 was included.

Likewise, the same fitting analysis previously described was used to optimize the value of *C*_pin_, which is required to perform measurements of an isolated load capacitance through the fast acquisition mode. In this case, through-hole capacitors of different sizes were used to bridge two I/O ports of the micro-controller, and Equation (4) was used as a fitting function for the measurements of the *N*_A0_ values shown in the Figure 9a. The *C*_pin_ values extracted for each micro-controller are shown in Table 2. The MATLAB scripts written to perform the OLS fitting are available in the Supplementary Materials.

## 3. Results

Commercial through-hole resistors and capacitors in the ranges 0.5 Ω–80 MΩ and 100 Ff–4.7 mF (±5%), respectively, were used to perform measurements of either isolated or in series and parallel electrical components with the AVR^®^ micro-controllers. The capacitances in the fF range were of SMD type (Kyocera AVX, Fountain Inn, SC, USA), and all capacitances above 1 μF were aluminum electrolyte capacitor type. The validations were made by comparing the measurements results with the components’ nominal values. In all cases, each data point corresponds to the average of 100 measurements performed in a continuous loop.

### 3.1. Characterization of Isolated Resistance Measurements

The resistance measurements were performed with a set of resistors independent of those used to linearize the ADC. Using the methodology described in Section 2.4, the voltages at port *P*_A0_ (*V*_A0_) were recorded with the ADC unit (*N*_A0_), and the measurements were inserted in Equation (2), to obtain the *R*_LOAD_ values shown in Figure 8b. This figure also includes the same resistances measurements recorded with a commercial instrument, the Fluke 8840A multimeter (Fluke Corporation, Everett, WA, USA), for reference and validation. These are excellent results for a low-cost technique. However, it is important to keep in mind that each individual point is the average of 100 consecutive measurements, performed in a loop. Table 3 provides numeric detail on the data shown in Figure 8b.

Figure 8c shows the relative accuracy (*a*_r_) of the measurements as a function of the nominal resistance, as defined in Section 2.11. Figure 8d shows their relative precision, *p*_r_, also defined in Section 2.11 (data points), and the estimated upper limit for the relative uncertainty caused by the digitalization round-off error, *u*_r_, defined in Equation (16) and represented by the dashed line. Note that the standard deviations are calculated on samples that are already averages of 100 points, which means that *u*_r_ should be divided by 10, for a correct comparison with *p*_r_. Both plots include white and grey shading regions to highlight the levels of *a*_r_ and *p*_r_ better than 5%, 10% and 20%. The two plots show better performance of the method in the intermediate resistance range and its degradation in the regime of very low or very high resistances. This is because the lower and upper limits for *R*_LOAD_ correspond to *N*_A0_ tending to the higher (*N*_max_) and lower (≈0) values of the digital output, respectively, where the roundoff errors introduced by digitalization become more important, affecting both precision and accuracy.

Most of the data points lay below the *u*_r_ curve. This is because *u*_r_ is merely an upper limit for the digitalization noise, which is actually lower. In any case, the plot indicates that the main uncertainty source in the determination of resistance is the digitalization roundoff.

In terms of performance, the ATmega32U4 delivers better results. The range defined by *p*_r_ < 5% is approximately 10 Ω–10 MΩ for the ATmega328P and 10 Ω–80 MΩ for the ATmega32U4, while the range defined by *a*_r_ < 5% is 100 Ω–100 kΩ for the ATmega328P and 100 Ω–10 MΩ for the ATmega32U4.

The sensitivity of the R-meter (minimum detectable increment in resistance) was assessed at a representative value of 1 kΩ, as well at increments of 0.5, 1 and 4.7 Ω. 50 measurements were acquired at each resistance value. The average of the 50 ADC counts were plotted against the resistance values and a local slope ADC counts/Ω was determined. The standard deviations of the 4 measurements were also calculated and averaged to get a typical value. A conservative estimative of the sensitivity was then performed by calculating the increase in resistance needed to shift the ADC count by two standard deviations, which was about 1.2 Ω or 0.1% of the nominal value. Additionally, a student *t*-test analysis was performed by comparing all the 4 datasets of 50 measurements against each other to conclude that they were all different (*p* < 0.05). This suggested that even an increment of 0.5 Ω is enough to change the output of the R-meter, which is about 2 times less than the previous conservative estimate.

### 3.2. Characterization of Isolated Capacitance Measurements: Fast Acquisition Method

Using the methodology described in Section 2.5.1 with different commercial capacitors varying from 100 fF to 100 nF, measurements of the voltage at port *P*_A0_ (*V*_A0_) were recorded with the ADC unit (*N*_A0_) and shown in Figure 9a. Figure 9a also includes a green dashed line that represents the theoretical lines for the two micro-controllers (overlapped), obtained from Equation (3) with the fitted *C*_pin_ values shown in Table 2. The measured *N*_A0_ and fitted *C*_pin_ values were replaced in Equation (4) to determine the load capacitance (*C*_LOAD_), shown in Figure 9b. At the end of Section 3.2, Table 4 was included, providing numeric detail on the data shown in Figure 9b. For reference and validation, this plot also includes the measurements recorded with two commercial instruments, the BK Precision 890C capacitance meter (B&K Precision Corporation, Yorba Linda, CA, USA) and the Fluke PM6304 impedance meter (Fluke Corporation, Everett, WA, USA) at the lowest frequency available (*f* = 50 Hz). The green dashed line is *C*_LOAD_ = *C*_nominal_, showing that the *C*_LOAD_ values determined by both micro-controllers match the target values within the range 100 Ff–10 nF, thus achieving a range of about 5 orders of magnitude. 

Figure 9c,d allow a more detailed view of the quality of this match and display *a*_r_, *p*_r_ and *u*_r_ (in the same way as in Figure 8c,d). The accuracy *a*_r_ drops significantly above 10 nF because the voltage drop at the load capacitance tends towards the circuit voltage source (*N*_A0_ → *N*_max_), as predicted by Equation (5), inducing large errors in the determination of *C*_LOAD_.

This is evidenced in the *u*_r_ curve represented (black dashed line in Figure 9d), showing the same V-shaped distribution *u*_r_, as described in Figure 8d, for the resistance measurement. The overlap between *u*_r_ and *p*_r_ means that the main source of variability is the digitalization noise, to which the fast C-meter adds almost no contribution.

The sensitivity estimates were performed according to the same lines described in Section 3.1, this time for the representative values of 18 pF and 22 pF, with small increments of 1, 1.2 and 1.5 pF. The sensitivity was estimated to be about 10–20 fF in both cases. The student *t*-test analysis also concluded that all the capacitance measurement datasets were different from each other (*p* < 0.05).

### 3.3. Characterization of Isolated Capacitance Measurements: Transient Acquisition Method

**Figure 10:**
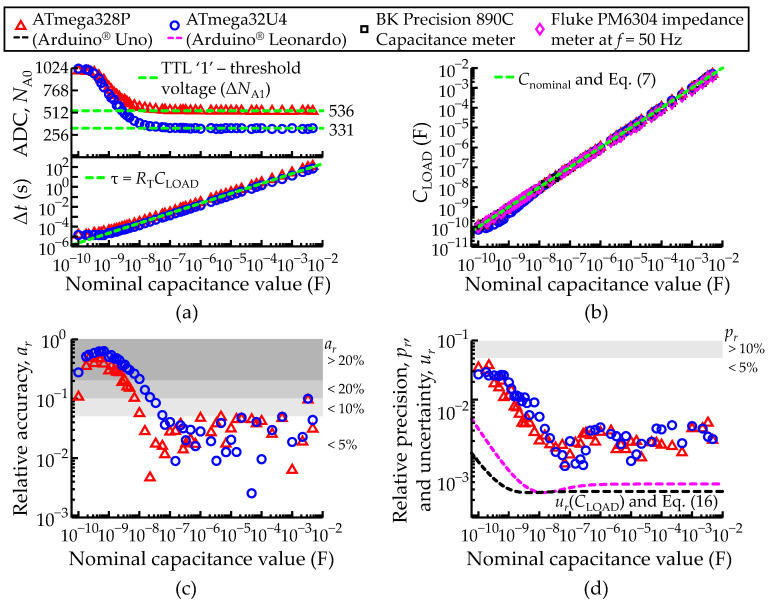
Comparison between the ATmega328P and ATmega32U4 AVR^®^ micro-controllers configured to record an isolated load capacitance (*C*_LOAD_) through the transient acquisition mode. (**a**) Measured ADC value and time (Δ*t*) until the TTL unit changes to digital state high, logic ‘1’ at port *P*_A0_ (*N*_A0_). Each ADC and Δ*t* sample consist of an average of 100 consecutive measurements. (**b**) Measured load capacitance (*C*_LOAD_) given by Equation (7). The green dashed lines represent the theoretical lines. (**c**) Relative accuracy (*a*_r_) and (**d**) relative precision (*p*_r_) of the measurements in function of *C*_nominal_. The black dashed line represents the relative uncertainty (*u*_r_) of the *C*_LOAD_ measurements according to Equation (16). The white and grey shading areas highlight the levels of *u*_r_, *a*_r_ and *p*_r_ better than 5%, 10% and 20%. A legend to describe the color scheme used in all plots of Figure 10 was included.

The capacitance meter in the transient mode was tested according to the methodology described in Section 2.5.2 with different commercial capacitors ranging from 100 pF to 4.7 mF. The transient acquisition mode requires waiting until the TTL logic unit returns ‘1’ and the elapsed time (Δ*t*) until that transition. The measurements of port *P*_A0_ (*N*_A0_), taken at the transition and the corresponding elapsed time (Δ*t*) are shown in Figure 10a. 

The upper graph shows *N*_A0_ as symbols. Note that by definition these *N*_A0_ readings correspond to TTL parameter *V*_IH_—High-Level Input Voltage (because they are acquired at the transition). The same graph shows that the lower the capacitance, the higher the *V*_IH_ of the TTL unit. This aspect is consistent with the expected operation mode of the TTL unit. In fact, the TTL unit uses the output high-level current (*i*_HL_) as a test condition, thence, as *C*_LOAD_ → *C*_pin_, the more leakage current flows through *C*_LOAD_, leading to a non-constant *V*_IH_. It should be remarked that the inconstancy of the threshold does not represent a problem for the application of Equation (7), since it only requires a given time and the associated reading *V*_A0_.

*V*_IH_ stabilizes above 22 nF because in that range *i*_HL_ remains essentially undisturbed. This allows the estimation of the “basal” *V*_IH_ (Δ*N*_A0_) averaging the values of *N*_A0_ at the transition for all samples above 22 nF, specifically Δ*N*_A0_ ≅ 536 for the ATmega328P and Δ*N*_A0_ ≅ 331 for the ATmega32U4, which are represented by the green dashed lines. The measured values of Δ*N*_A0_ are consistent with the typical values described in Table 1. 

The measured elapsed time (Δ*t*) shown in the bottom graph of Figure 10a closely matches the green dashed line, which represents the characteristic time constant *τ* = *R*_T_*C*_LOAD_, where *R*_T_ = *R*_AIN_ || *R*_pu_ + *R*_out_. The linearity still holds in spite of a non-constant *V*_IH_ due to the role of *i*_HL_, since the leakage current is also determined by the RC constant of the circuit. The RT values differ only slightly for the two micro-controllers (*R*_T_ ≅ 35 kΩ for ATmega328P and *R*_T_ ≅ 37 kΩ for ATmega32U4), causing overlap of the corresponding time constant lines.

For the smallest capacitances (*C*_LOAD_ < 1 nF), Δt tends toward a plateau (Δ*t*_min_) given by 12.5 μs for both micro-controllers, because *C*_LOAD_ → *C*_pin_. Thus, the *C*_LOAD_ values determined with Equation (7), and shown in Figure 10b, are affected by larger errors in the low capacitance range. This is not very apparent in this figure, where the data points seem very close to the green dashed line (*C*_LOAD_ = *C*_nominal_) because of the logarithmic scale. Still, at the end of Section 3.3, Table 5 provides numeric detail on the data shown in Figure 10b.

The plots of *a*_r_ in Figure 10c illustrate better the difficulties in the low capacitance ranges. The relative accuracy drops significantly (worse than 5%) below 70 nF for the ATmega32U4 and below 10 nF for the ATmega328P. However, both remain, at least, on a 5% accuracy level above those critical values, representing a linear response of the instrument across 6 decades.

Figure 10d shows *p*_r_ (points) and *u*_r_ (dashed lines). Contrary to the fast C-meter case, here the *u*_r_ and *p*_r_ lines are clearly above *u*_r_, which means that the method introduces sources of noise other than digitalization. This is probably because variations of some ADC units in the threshold level impact much more the measurement than one ADC unit only, related to the digitalization error. These variations may be caused by the internal noise of the micro-processors. The performance degradation in the low capacitance regime is also evident from this figure.

The sensitivity estimates were performed according to the same lines described in Section 3.1, for the representative value of 1 μF, with small increments of 1.8, 2.2, 2.7, 3.3, 3.9 and 4.7 nF. The sensitivity was estimated to be about 10–20 fF in both cases. The student *t*-test analysis also concluded that all the capacitance measurement datasets were different from each other (*p* < 0.05).

### 3.4. Characterization of Measurements for Serial RC Networks

**Figure 11:**
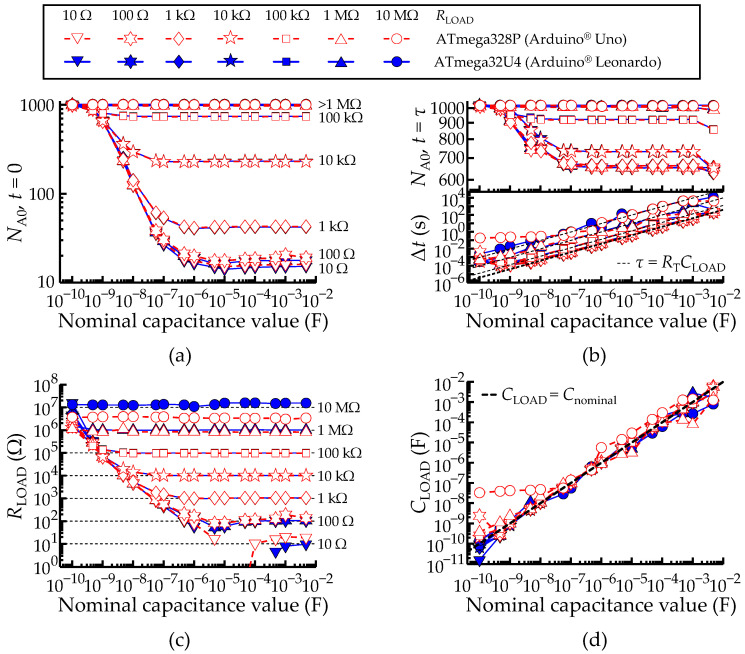
Comparison between the ATmega328P and ATmega32U4 AVR^®^ micro-controllers configured to measure a serial RC network. (**a**) Measured ADC value at *t =* 0. (**b**) Measured ADC value and time (Δ*t*) until *V*_A1_ ≥ *V*_C_. (**c**,**d**) Obtained *R*_LOAD_ and *C*_LOAD_ values according to Equations (2) and (10), respectively. A legend to describe the color scheme used in all plots of Figure 11 was included. The black dashed lines always represent the theoretical lines.

Using different sets of commercial resistors varying in factors of 10, from 10 Ω to 10 MΩ, and capacitors with 2 samples per order of magnitude, from 100 pF to 4.7 mF, seven trials were made to test the methodology described in Section 2.6. Each trial consisted of keeping the *R*_LOAD_ constant and varying the *C*_LOAD_. Figure 11 compiles a total of 14 trials carried out with the two micro-controllers. The white points with red edges represent the data measured with the ATmega328P, and the blue points represent the ATmega32U4. They are mostly overlapped.

The method implies measurement of the port *P*_A0_ voltage level (*V*_A0_) at *t* = 0 to find the offset voltage (*V*_R_) and at *t* = Δ*t* ≥ *τ*, to find the voltage level *V*_C_ (defined in Section 2.6). 

Figure 11a,b show the measured discrete values (*N*_A0_) at the instants *t* = 0 and *t* ≥ *τ*, respectively, with both micro-controllers. Figure 11b also includes in the bottom graph the measured elapsed time (Δ*t*) until *V*_A1_ ≥ *V*_C_, together with the theoretical time constants, *τ* = *R*_T_*C*_LOAD_. Note that, from Equation (9), *N*_A0_(*t* = 0) = *V*_R_, which is independent of *C*_LOAD_. This means that all the curves in Figure 11a should be horizontal lines. However, the minimum available acquisition time (ca. 3 μs) is insufficient to capture the initial curve values for *C*_LOAD_ < 1 μF. Thus, the captured *N*_A0_ values at *t* = 0 tend to *N*_max_ in the limit of very small capacitances. This explains why the (ideal) straight lines become distorted in Figure 11a, especially for lower *R*_LOAD_. Likewise, the plateau *N*_A0_ (*t* ≥ *τ*) = *V*_C_ is reached for any *C*_LOAD_ > 100 nF, which hampers the measurements of *N*_A0_ after *t* ≥ *τ* in this range and induces the same type of distortion in Figure 11b, top.

*R*_LOAD_ and *C*_LOAD_ were determined through the use of Equations (2) and (10), with the results shown in Figure 11c,d, respectively. The horizontal lines in Figure 11c represent the ideal result, *R*_measured_ = *R*_LOAD_. The measured *R*_LOAD_ values are unreliable within a domain in the R-C plane approximately defined by *R*_nominal_ *C*_nominal_ < 10^−4^ s.

As discussed above, the system loses accuracy for smaller values of *R*_LOAD_. In fact, when *R*_LOAD_ ≪ (*R*_AIN_ || *R*_pu_) + *R*_out_, then *R*_T_ = (*R*_AIN_ || *R*_pu_) + *R*_LOAD_ + *R*_out_ ≈ *R*_pu_ + *R*_out_, and the information about *R*_LOAD_ is lost. For this reason, the higher the *R*_LOAD_, the better the accuracy. This observation is valid for both micro-controllers, except for the trials performed with a *R*_LOAD_ of 10 MΩ since *N*_A0_ → *N*_max_ at *t* = 0.

The measurements of the elapsed time (Δ*t*), after *V*_A0_ ≥ *V*_C_, shown in the bottom graph of Figure 11b, exhibit a relative shift in the vertical axis due to the different *R*_LOAD_ values. Deviations from the straight line are consequence of the excess digitalization uncertainty. This aspect is evidenced after determination of the *C*_LOAD_ values using Equation (10) and shown in Figure 11d, where the deviations from the black dashed line (*C*_LOAD_ = *C*_nominal_) relate to the excess digitalization uncertainty.

Overall, there is a trade-off between the *R*_LOAD_ and *C*_LOAD_ ranges for best accuracy. There are three extreme regimes: (case 1) *R*_LOAD_ is large (>1 MΩ), irrespective of the *C*_LOAD_ value: the accuracy of the measurements is good for the resistance and poor for the capacitance; (case 2) small *R*_LOAD_ (<100 kΩ) and large *C*_LOAD_ (>1 nF): measurements with poor accuracy for the resistance, good for the capacitance; (case 3) small *R*_LOAD_ (<100 kΩ) and small *C*_LOAD_ (<0.1 nF): measurements with very poor accuracy for the resistance, poor for the capacitance. Outside these extreme regimes, the accuracy is at least acceptable for *R*_LOAD_ and *C*_LOAD_ simultaneously.

### 3.5. Characterization of Measurements for Parallel RC Networks

The experimental procedures to perform the characterization of measurements for parallel RC networks were the same as those of the previous section.

For the parallel RC, the saturation voltage (*V*_F_) lies below *V*_S_, and it is necessary to determine both *V*_F_ and *τ* from the data. There are simple and computationally light algorithms allowing the identification of a stationary plateau, such as that occurring at *V*_F_. These have been tested and verified, but including here the description of such methods would increase the length of this report. Thus, the subsequent analysis assumes that *τ* is already known. No generality is lost with this assumption.

Therefore, to each combination of a parallel RC network (*R*_LOAD_ || *C*_LOAD_) that was measured, the time constant *τ* was directly assumed as *R*_T_*C*_LOAD_. The total acquisition time was set to 10*τ*, *V*_F_ was read from *V*_A0_ at *t* = 5*τ* and *V*_C_ was read from *V*_A0_ at *t* = *τ*.

**Figure 12:**
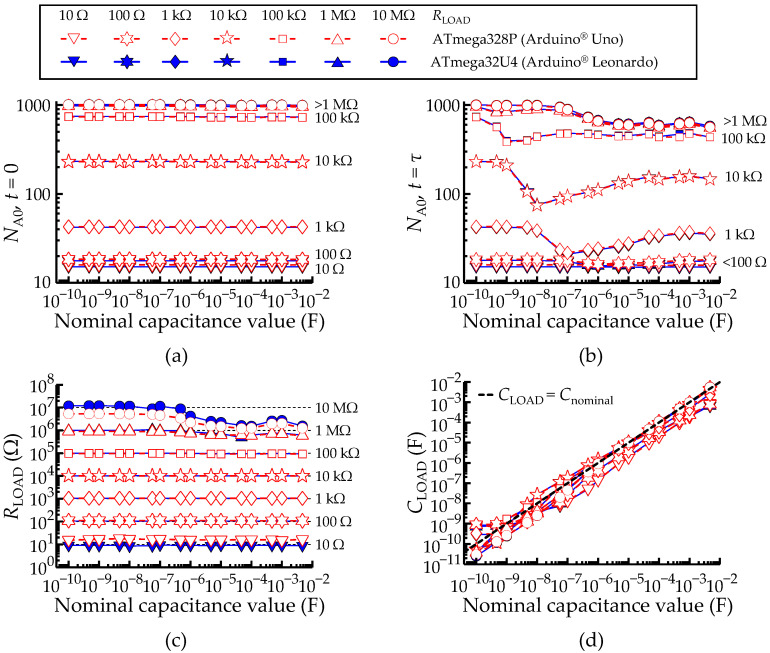
Comparison between the ATmega328P and ATmega32U4 AVR^®^ micro-controllers configured to measure a parallel RC network. (**a**) Measured ADC value at *t =* 5*τ*. (**b**) Measured ADC value at *t = τ*. (**c**,**d**) Obtained *R*_LOAD_ and *C*_LOAD_ values according to Equations (2) and (12), respectively. A legend to describe the color scheme used in all plots of Figure 12 was included. The black dashed lines always represent the theoretical lines.

Figure 12 aligns the results in two columns. The left column [(a) and (c)] refers to the measurements of *R*_LOAD_ values and the right one [(b) and (d)] refers to the measurements of *C*_LOAD_ values. The *N*_A0_ values at the instants *t* = 5*τ* and *t* = *τ* are shown in Figure 12a,b, respectively. Figure 12c,d show the *R*_LOAD_ and *C*_LOAD_ values, which were obtained from Equations (2) and (12), respectively.

The *R*_LOAD_ values are generally in line with the measurements carried on with isolated resistors shown in Figure 8b, but with few deviations to linearity (at 10 Ω and any *C*_LOAD_, for the ATmega328P; at 1 MΩ and *C*_LOAD_ > 1 μF, for both micro-controllers).

As discussed before, in Section 3.4, a too large minimal acquisition time and/or the excess of digitalization noise are the reason the collected samples deviate from the theoretical lines represented by the horizontal black dashed lines. Similarly, the *C*_LOAD_ values are in line with the measurements carried on with isolated capacitors shown in Figure 9b. However, the accuracy of the measurements is variable, for the same reasons mentioned above.

Overall, there is a trade-off between the *R*_LOAD_ and *C*_LOAD_ ranges for best accuracy. There are two extreme regimes: (case 1) *C*_LOAD_ is small (<1 μF), and *R*_LOAD_ is large (<1 kΩ): the accuracy of the measurements is good for the resistance and poor for the capacitance; (case 2) large *R*_LOAD_ (>1 MΩ) and small *C*_LOAD_ (<1 μF): measurements with very poor accuracy for the capacitance, and poor for the resistance. Outside these extreme regimes, the accuracy is at least acceptable for *R*_LOAD_ and *C*_LOAD_ simultaneously.

## 4. Discussion and Conclusions

This work described and characterized methods to accurately measure impedance using Arduino^®^ boards with built-in AVR^®^ micro-controllers. This is highly remarkable considering the ultra-low cost of the hardware. The measurement method allows the extraction of the resistance (R) and capacitance (C) values of either isolated or series and parallel configuration. Furthermore, inductance (L) measurements can be also performed, yet the range of measurable values is not very useful.

To check the cross-platform applicability of our proposed RCL-meter, two different AVR^®^ micro-controllers assembled on Arduino^®^ boards were selected, namely the ATmega328P assembled on an Arduino^®^ Uno and the ATmega32U4 assembled on an Arduino^®^ Leonardo. A benchmark was made to test the performance of micro-processors.

As for the measurements of isolated resistances and capacitances, the ATmega32U4 outperforms the ATmega328P, and some specific differences were identified. For instance, the ATmega32U4 was revealed to be more efficient in terms of acquisition time, providing a significant improvement when recording long-term transients. In the worst-case scenario, when recording an isolated capacitance (*C*_LOAD_ = 4.7 mF) through the transient acquisition mode, the ATmega32U4 performs two times faster than the ATmega328P. In addition, when measuring both *C*_LOAD_ and *R*_LOAD_ values of in-series or parallel RC networks, the ATmega32U4 takes a slight advantage over the ATmega328P for larger values of *R*_LOAD_, while, conversely, the ATmega32U4 performs better for larger values of *C*_LOAD_.

The noise profiles of the direct measurements (R-meter and fast C-meter) are essentially defined by the digitalization noise, while the transient C-meter brings an important extra noise source from the variability in the determination of instants of time required to perform the calculations.

The fast and transient C-meter methods complement each other, since the fast method is best for low capacitances and the transient method best for high capacitances. Operated together, they are able to deliver a relative accuracy equal to or better than 20% in the range 100 fF–10 mF, that is, across 11 orders of magnitude. Furthermore, the accuracy is equal to or better than 5% in the ranges 100 fF–100 pF and 100 nF–10 mF. The series and parallel RC combinations are also able to deliver good measurements of R and C in specific domains of the R-C plane.

Additional investigations were made to analyze the performance of both Arduino^®^ boards supplied via a large power bank (Litionite Tanker 90 W/50,000 mAh) and using a data logger shield (RobotDyn^TM^, Zhuhai, China) to store the measurements in a local micro-SD card. This work led to the conclusion that no substantial improvements are achieved by using a low-noise and low-uncertainty voltage supply, and therefore all presented data considers the typical noise and uncertainty from a common USB interface voltage supply source. However, the voltage supply unit determines the overall measurement quality in regions close to the ADC unit threshold values (*N*) for *N* < 20 or *N* > 1000.

Additionally, measurements of the same *R*_LOAD_ and *C*_LOAD_ values were made with commercial instruments and presented in the manuscript to provide insight on the overall performance of the ATmega328P and ATmega32U4 micro-controllers as a low-cost alternative to more expensive and sophisticated instruments.

Moreover, the concept proposed in this work, based on AVR^®^ micro-controllers, may possibly be extended to other microcontrollers such as the STM32 family based on ARM architecture. The latter have more or improved integrated hardware features relative to the former, such as more flash memory, higher resolution ADC drive and faster clocks. On the other hand, using more sophisticated microcontrollers brings the disadvantage of idle but power-consuming internal hardware, for example, the digital-to-analog unit (DAC), which is not required in the present work. In any case, the inherent specificities of each architecture imply different implementations, difficult to cover in a single report.

The work carried out in the investigation of an ultra-low-cost RCL meter was mainly targeted towards impedimetric biosensor measurements, in order to facilitate the integration of the sensing and processing layers to the IoT. Its simplicity opens new possibilities for the improvement of ongoing and future projects in the field of smart sensing. The long-term goal of this work is to integrate the control of the sensing and processing layers into the Web of Things (WoT), which is an upper layer of interaction between devices that may be managed by artificial intelligence.

## Figures and Tables

**Table 1:** Typical values of the operational voltage of the circuits (*V*_S_), the ADC unit, the internal circuitry to each I/O port and the TTL unit to each AVR^®^ micro-controller [26,27].

Voltage Source	ADC Unit		Internal Circuitry Parameters				TTL Unit			
							ATmega328P		ATmega32U4	
***V*_S_** (V)	***n***	***N*_max_** (2*^n^* − 1)	***R*_AIN_** (MΩ)	***R*_out_** (Ω)	***R*_pu_** (kΩ)	***C*_pin_** (pF)	***V*_IL_** (V)	***V*_IH_** (V)	***V*_IL_** (V)	***V*_IH_** (V)
5	10	1023	100	600	32	24	−0.5–1.5	3.0–5.5	−0.5–0.9	1.9–5.5

**Table 2:** Optimized values of the *R*_AIN_, *R*_out_, *R*_pu_ and *C*_pin_ for both ATmega328P and ATmega32U4 AVR^®^ micro-controllers.

ATmega328P				ATmega32U4			
***R*_AIN_** (MΩ)	***R*_pu_** (kΩ)	***R*_out_** (Ω)	***C*_pin_** (pF)	***R*_AIN_** (MΩ)	***R*_pu_** (kΩ)	***R*_out_** (Ω)	***C*_pin_** (pF)
3.537	36.89	565.8	23.48	5.451	36.66	542.2	25.5

**Table 3:** Comparison of the ATmega328P, ATmega32U4 and Fluke 8840A measured load resistance (*R*_LOAD_) values ± standard deviation (SD).

*R*_nominal_	ATmega328P	ATmega32U4	Fluke 8840A		ATmega328P	ATmega32U4	Fluke 8840A
	*R*_LOAD_ ± SD			*R*_nominal_	*R*_LOAD_ ± SD		
0.5 Ω	0.6 ± 0.4	0.62 ± 0.4	0.548 ± 0.001	5.6 kΩ	5.679 ± 0.001	5.7261 ± 0.0006	5.5390 ± 0.0002
1 Ω	1.2 ± 0.2	0.86 ± 0.2	1.044 ± 0.001	8.2 kΩ	8.539 ± 0.009	8.6078 ± 0.0009	8.1837 ± 0.0002
2.2 Ω	1.6 ± 0.3	2.30 ± 0.5	2.247 ± 0.001	10 kΩ	10.101 ± 0.002	10.1772 ± 0.0008	9.9313 ± 0.0002
5.6 Ω	5.7 ± 0.4	5.70 ± 0.6	5.645 ± 0.001	22 kΩ	21.9 ± 0.3	22.1858 ± 0.0008	21.846 ± 0.0000
8.2 Ω	9.1 ± 0.2	7.72 ± 0.6	8.251 ± 0.001	56 kΩ	55.0 ± 0.1	55.789 ± 0.002	55.8529 ± 0.0003
10 Ω	10.5 ± 0.3	8.77 ± 0.5	10.031 ± 0.005	82 kΩ	80.8 ± 0.1	81.891 ± 0.002	81.914 ± 0.002
22 Ω	22.4 ± 0.4	20.1 ± 0.5	21.901 ± 0.002	100 kΩ	97.3 ± 0.2	98.812 ± 0.003	98.622 ± 0.008
56 Ω	59.7 ± 0.4	55.7 ± 0.5	56.023 ± 0.004	220 kΩ	211.9 ± 0.4	217.55 ± 0.01	219.267 ± 0.007
82 Ω	83.3 ± 0.3	83.1 ± 0.4	82.532 ± 0.0014	560 kΩ	535 ± 5	549.7 ± 0.1	561.045 ± 0.009
100 Ω	102.2 ± 0.3	101.9 ± 0.4	99.44 ± 0.02	820 kΩ	777 ± 7	811.4 ± 0.2	824.71 ± 0.01
220 Ω	224.3 ± 0.3	224.2 ± 0.4	220.560 ± 0.004	1 MΩ	0.906 ± 0.007	0.9894 ± 0.0002	1.0140 ± 0.00004
560 Ω	576.9 ± 0.4	573.6 ± 0.8	556.14 ± 0.01	2.2 MΩ	2.14 ± 0.05	2.193 ± 0.004	2.2171 ± 0.0002
820 Ω	835.8 ± 0.6	833.840 ± 0.0001	817.19 ± 0.03	6.8 MΩ	6.3 ± 0.3	6.89 ± 0.01	6.986 ± 0.002
1 kΩ	1.0348 ± 0.0006	1.0331 ± 0.0004	0.9945 ± 0.0001	8.2 MΩ	8.8 ± 0.3	7.87 ± 0.05	8.2066 ± 0.0001
2.2 kΩ	2.242 ± 0.001	2.2596 ± 0.0005	2.1955 ± 0.0001	10 MΩ	10.3 ± 0.2	9.60 ± 0.03	10.129 ± 0.008

**Table 4:** Comparison of the ATmega328P, ATmega32U4 and Fluke 8840A measured load capacitance (*C*_LOAD_) values ± standard deviation (SD) through the fast acquisition mode.

*C*_nominal_	ATmega-328P	ATmega-32U4	Fluke PM6304	BK 890C		ATmega-328P	ATmega-32U4	Fluke PM6304	BK 890C
	*C*_LOAD_ ± SD				*C*_nominal_	*C*_LOAD_ ± SD			
1 pF	1.126 ± 0.004	1.050 ± 0.005	12 ± 8	1.3 ± 0.6	560 pF	550.0 ± 0.8	510.89 ± 0.05	526 ± 3	531 ± 3
1.5 pF	1.640 ± 0.002	1.541 ± 0.003	16 ± 18	1.9 ± 0.3	680 pF	644 ± 1	600.30 ± 0.05	678 ± 5	677 ± 7
2.7 pF	2.834 ± 0.003	2.782 ± 0.004	14 ± 8	3.3 ± 0.6	1 nF	1.012 ± 0.003	0.93558 ± 0.00004	0.999 ± 0.009	1.008 ± 0.004
3.9 pF	4.101 ± 0.003	3.998 ± 0.004	12 ± 5	8 ± 1	1.5 nF	1.445 ± 0.007	1.34302 ± 0.00003	1.493 ± 0.005	1.517 ± 0.002
5.8 pF	5.860 ± 0.002	5.709 ± 0.003	17 ± 19	7 ± 3	2.2 nF	2.08 ± 0.01	1.88744 ± 0.00004	2.185 ± 0.005	2.24 ± 0.01
8.2 pF	8.527 ± 0.003	8.278 ± 0.003	17 ± 5	9.3 ± 0.4	3.3 nF	3.29 ± 0.03	2.93701 ± 0.00003	3.314 ± 0.006	3.401 ± 0.003
10 pF	10.231 ± 0.002	9.900 ± 0.002	18 ± 5	11.2 ± 0.3	6.8 nF	7.4 ± 0.2	6.58318 ± 0.00003	7.096 ± 0.02	7.13 ± 0.06
20 pF	20.711 ± 0.003	19.672 ± 0.002	25 ± 4	22.300 ± 0.000	7.5 nF	9.0 ± 0.2	7.98201 ± 0.00004	7.56 ± 0.05	7.7540 ± 0.0004
47 pF	46.934 ± 0.009	46.59 ± 0.04	49 ± 7	49.3 ± 0.5	10 nF	12.0 ± 0.4	9.51585 ± 0.00003	10.15 ± 0.07	9.668 ± 0.001
82 pF	80.88 ± 0.02	81.01 ± 0.02	89 ± 7	83.4 ± 0.5	15 nF	20 ± 1	17.8541 ± 0.00004	15.44 ± 0.06	15.570 ± 0.0000
100 pF	99.56 ± 0.04	100.87 ± 0.03	99 ± 5	103.183 ± 0.04	22 nF	27 ± 1	28.6855 ± 0.00003	21.623 ± 0.007	22 ± 1
180 pF	179.1 ± 0.1	178.6 ± 0.2	187 ± 1	185 ± 1	56 nF	46 ± 5	70.0663 ± 0.00002	57.21 ± 0.04	57.2 ± 0.9
220 pF	210.7 ± 0.2	208.5 ± 0.2	221 ± 12	217.6 ± 0.5	68 nF	47 ± 5	75.7749 ± 0.00002	68.39 ± 0.07	69 ± 2
470 pF	440.0 ± 0.7	409.77 ± 0.05	469 ± 6	449.9 ± 0.8	100 nF	55 ± 5	96.2257 ± 0.00003	101.3 ± 0.02	101.536 ± 0.005

**Table 5:** Comparison of the ATmega328P, ATmega32U4 and Fluke 8840A measured load capacitance (*C*_LOAD_) values ± standard deviation (SD) through the transient acquisition mode.

*C*_nominal_	ATmega-328P	ATmega-32U4	Fluke PM6304	BK 890C		ATmega-328P	ATmega-32U4	Fluke PM6304	BK 890C
	*C*_LOAD_ ± SD				*C*_nominal_	*C*_LOAD_ ± SD			
100 pF	111 ± 4	72 ± 1	99 ± 5	101.41 ± 0.04	2.2 μF	2.259 ± 0.007	2.158 ± 0.006	2.2742 ± 0.0004	2.292 ± 0.005
1 nF	0.703 ± 0.008	0.49 ± 0.01	0.999 ± 0.009	1.008 ± 0.004	4.7 μF	4.933 ± 0.008	4.883 ± 0.009	4.24 ± 0.04	4.5010 ± 0.0006
2.2 nF	1.591 ± 0.009	1.22 ± 0.01	2.185 ± 0.005	2.24 ± 0.01	6.8 μF	6.92 ± 0.01	6.71 ± 0.02	7.1687 ± 0.0001	7.1870 ± 0.0001
4.7 nF	3.99 ± 0.02	3.26 ± 0.02	4.77 ± 0.03	4.90 ± 0.03	10 μF	10.31 ± 0.02	10.204 ± 0.009	9.76 ± 0.01	9.998 ± 0.02
6.8 nF	6.10 ± 0.02	5.01 ± 0.05	7.10 ± 0.02	7.13 ± 0.06	22 μF	23.01 ± 0.02	23.06 ± 0.04	20.98 ± 0.03	21.29 ± 0.03
10 nF	9.43 ± 0.02	7.85 ± 0.04	10.15 ± 0.07	9.667 ± 0.001	47 μF	49.10 ± 0.09	47.12 ± 0.09	42.69 ± 0.04	43.0 ± 0.5
22 nF	22.10 ± 0.04	19.66 ± 0.04	21.623 ± 0.007	22 ± 1	68 μF	70.6 ± 0.1	70.8 ± 0.2	66.39 ± 0.02	68.68 ± 0.06
56 nF	56.89 ± 0.09	53.03 ± 0.06	57.21 ± 0.04	57.2 ± 0.9	100 μF	104.2 ± 0.2	101.0 ± 0.3	98.14 ± 0.04	102.30 ± 0.08
68 nF	67.22 ± 0.06	65.52 ± 0.05	68.39 ± 0.07	69 ± 2	220 μF	225.6 ± 0.4	226.5 ± 0.8	200.29 ± 0.08	206.7 ± 2
100 nF	97.2 ± 0.1	95.9 ± 0.2	101.34 ± 0.02	101.536 ± 0.005	470 μF	491.9 ± 0.6	446.6 ± 0.8	455.7 ± 0.2	472.6 ± 3
220 nF	228.3 ± 0.4	212.2 ± 0.2	221.79 ± 0.03	221.900 ± 0.000	1 mF	1.006 ± 0.003	0.981 ± 0.004	0.9608 ± 0.0006	0.994 ± 0.006
470 nF	480 ± 1	456 ± 1	468.54 ± 0.05	469.800 ± 0.000	2.2 mF	2.240 ± 0.007	2.250 ± 0.007	2.1667 ± 0.0005	2.1987 ± 0.0000
680 nF	692 ± 1	669 ± 2	665.67 ± 0.2	680.000 ± 0.000	3.3 mF	3.62 ± 0.02	3.631 ± 0.009	3.1891 ± 0.0007	3.441 ± 0.002
1 μF	1.047 ± 0.003	0.972 ± 0.003	0.983 ± 0.001	0.989 ± 0.007	4.7 mF	4.85 ± 0.01	4.91 ± 0.01	4.6754 ± 0.0005	4.935 ± 0.003

## Data Availability

Not applicable.

## Supplementary Materials

The following supporting information can be downloaded at: https://www.mdpi.com/article/10.3390/s22062227/s1, C++ code A.1: High-level routine to measure isolated resistances; C++ code A.2: High-level routine to measure isolated capacitances through the fast acquisition mode; C++ code A.3: High-level routine to measure isolated capacitances through the transient acquisition mode; C++ code A.4: High-level routine to measure a series RC-network; C++ code A.5: High-level routine to measure a parallel RC-network; C++ code A.6: High-level routine to measure an isolated inductance; MATLAB code B.1: High-level routine for fitting the Ordinary Least Squares (OLS) function to the resistance values; MATLAB code B.2: High-level routine for fitting the Ordinary Least Squares (OLS) function to the capacitance values.
