# Supplementary material for: An Ultra-Low-Cost RCL-Meter
Source: Sensors (Basel). 2022 Mar 14;22(6):2227. doi: 10.3390/s22062227 (PMC8950037; doi:10.3390/s22062227)
Supplement: Supplementary file 1 [file sensors-22-02227-s001.zip › sensors-1582127-supplementary.pdf]

Supplementary Materials

A.1 – High-level routine to measure isolated resistances in C++ language

```
// Arduino built-in an isolated resistance meter according to Section 2.4

#include <SPI.h>

// Assign analog pin A0 to port PA0 at eq. circuit shown in Figure 1(f)
#define PA0  A0
// Define analog pin A3 to port PA1 at eq. circuit shown in Figure 1(f)
#define PA1  A3
// Maximum ADC value considering the 10-bit (n) of resolution: 2^(n)-1
#define NMAX 1023.0
// Analog input resistance value [Ohm(Omega)], see Table 2
#define RAIN 5.451E+06
// Pullup resistance value [Ohm(Omega)], see Table 2
#define RPU  36.66E3
// Output resistance value [Ohm(Omega)], see Table 2
#define ROUT 542.2
// BaudRate of the serial interface. Typical value is 9600, and up to 2E6
#define RxTx 2E6

void setup() {
  // Start serial monitor
  Serial.begin( RxTx );

  // Set-up port PA0 to low output
       pinMode( PA0, OUTPUT );
  digitalWrite( PA0,    LOW );

  // Set-up port PA1 to low output
       pinMode( PA1, OUTPUT );
  digitalWrite( PA1,    LOW );

  // Force the discharge of all capacitances
  delay( 1000 );

  // Set-up port PA0 to pull-up input
  pinMode( PA0, INPUT_PULLUP );

  // Wait 1 ms to ensure stray capacitances are fully charged
  delay(1);
}

void loop() {

  // Measure the discrete value of the analog port PA0
  float NA0 = analogRead( PA0 );

  // Calculate variable K using Eq.(2)
  float K = ( RPU * NA0 ) / ( NMAX - NA0 );

  // Calculate load resistance (R_LOAD) using Eq.(2)
  float R_LOAD = ( ( RAIN + ROUT ) * K - RAIN * ROUT ) / ( RAIN - K );

  // Print resistance value to the serial interface
  Serial.print( F( "Resistance: " ) );
  if( R_LOAD >= 1E6 ) {
```

```
    Serial.print( R_LOAD * 1E-6 , 8 );
    Serial.print( F( " M" ) );
  } else if( R_LOAD >= 1E3 ) {
    Serial.print( R_LOAD * 1E-3 , 8 );
    Serial.print( F( " k" ) );
  } else {
    Serial.print( R_LOAD , 8 );
    Serial.print( F( " " ) );
  }
  Serial.println( F( "Ohm" ) );
}
```

A.2 – High-level routine to measure isolated capacitances through the fast acquisition mode in C++ language

```
// Arduino built-in an isolated capacitance meter through the fast
// acquisition mode, and according to Section 2.5.1

#include <SPI.h>

// Assign analog pin A0 to port PA0 at eq. circuit shown in Figure 3(a)
#define PA0  A0
// Define analog pin A3 to port PA1 at eq. circuit shown in Figure 3(a)
#define PA1  A3
// Maximum ADC value considering the 10-bit (n) of resolution: 2^(n)-1
#define NMAX 1023.0
// Stray capacitance [F(Farad)], see Table 2
#define CPIN 25.55E-12
// BaudRate of the serial interface. Typical value is 9600, and up to 2E6
#define RxTx 2E6

void setup() {
  // Start serial monitor
  Serial.begin( RxTx );

  // Set-up port PA0 to low output
       pinMode( PA0, OUTPUT );
  digitalWrite( PA0,    LOW );

  // Set-up port PA1 to low output
       pinMode( PA1, OUTPUT );
  digitalWrite( PA1,    LOW );
  // Force the discharge of all capacitances
  delay( 1000 );
}

void loop() {
  // Set-up port PA0 to input
       pinMode( PA0 , INPUT );

  // Set-up port PA1 to high output
  digitalWrite( PA1 ,  HIGH );

  // Measure the discrete value of the analog port PA0
  float NA0 = analogRead( PA0 );

  // Set-up port PA1 to low output
  digitalWrite( PA1 ,   LOW );
```

```
  // Set-up port PA0 to low output
       pinMode( PA0, OUTPUT );
  digitalWrite( PA0,    LOW );

  // Calculate load capacitance (C_LOAD) using Eq.(4)
  float C_LOAD = CPIN * NA0 / ( NMAX - NA0 );

  // Print capacitance value to the serial interface
  Serial.print( F( "Capacitance: " ) );
  if( C_LOAD < 1E-12 ) {
    Serial.print( C_LOAD * 1E15 , 8 );
    Serial.print( F( " f" ) );
  } else if( C_LOAD < 1E9 ) {
    Serial.print( C_LOAD * 1E12 , 8 );
    Serial.print( F( " p" ) );
  } else if( C_LOAD < 1E6 ) {
    Serial.print( C_LOAD * 1E9 , 8 );
    Serial.print( F( " n" ) );
  } else if( C_LOAD < 1E3 ) {
    Serial.print( C_LOAD * 1E6 , 8 );
    Serial.print( F( " u" ) );
  } else if( C_LOAD < 1E0 ) {
    Serial.print( C_LOAD * 1E3 , 8 );
    Serial.print( F( " m" ) );
  } else {
    Serial.print( C_LOAD , 8 );
    Serial.print( F( " " ) );
  }
  Serial.println( F( "F" ) );
}
```

A.3 – High-level routine to measure isolated capacitances through the transient acquisition mode in C++ language

```
// Arduino built-in an isolated capacitance meter through the transient
// acquisition mode, and according to Section 2.5.2

#include <SPI.h>

// Assign analog pin A0 to port PA0 at eq. circuit shown in Figure 1(f)
#define PA0  A0
// Define analog pin A3 to port PA1 at eq. circuit shown in Figure 1(f)
#define PA1  A3
// Maximum ADC value considering the 10-bit (n) of resolution: 2^(n)-1
#define NMAX 1023.0
// Analog input resistance value [Ohm(Omega)], see Table 2
#define RAIN 5.451E+06
// Pullup resistance value [Ohm(Omega)], see Table 2
#define RPU  36.66E3
// Output resistance value [Ohm(Omega)], see Table 2
#define ROUT 542.2
// BaudRate of the serial interface. Typical value is 9600, and up to 2E6
#define RxTx 2E6

void setup() {
  // Start serial monitor
  Serial.begin( RxTx );
```

```
  // Set-up port PA0 to low output
       pinMode( PA0, OUTPUT );
  digitalWrite( PA0,    LOW );

  // Set-up port PA1 to low output
       pinMode( PA1, OUTPUT );
  digitalWrite( PA1,    LOW );

  // Force the discharge of all capacitances
  delay( 1000 );
}

void loop() {
  // Set-up port PA1 to input pull-up
  pinMode( PA0 , INPUT_PULLUP );

  // Read timer in microseconds (us)
  unsigned long TS = micros();

  // Wait until TTL unit return logic '1'
  float NA0 = 0;
  do {
      NA0 = digitalRead(PA0);
  } while (NA0 < 1.0);

  // Read timer in microseconds (us)
  unsigned long TE = micros();

  // Measure the discrete value of the analog port PA0
  NA0 = analogRead( PA0 );

  // Set-up port PA0 to low output
       pinMode( PA0, OUTPUT );
  digitalWrite( PA0,    LOW );

  // Calculate elapsed time, Delta_t in seconds (s)
  float T = ( (float)( TE / 1000L ) - (float)( TS / 1000L ) ) / 1000;

  // Calculate total resistance (RT) value
  float RT = ROUT + ( RAIN * RPU ) / ( RAIN + RPU );

  // Calculate load capacitance (C_LOAD) using Eq.(7)
  float C_LOAD = T / ( RT * log( NMAX / ( NMAX - NA0 ) ) );

  // Print capacitance value to the serial interface
  Serial.print( F( "Capacitance: " ) );
  if( C_LOAD < 1E-12 ) {
    Serial.print( C_LOAD * 1E15 , 8 );
    Serial.print( F( " f" ) );
  } else if( C_LOAD < 1E9 ) {
    Serial.print( C_LOAD * 1E12 , 8 );
    Serial.print( F( " p" ) );
  } else if( C_LOAD < 1E6 ) {
    Serial.print( C_LOAD * 1E9 , 8 );
    Serial.print( F( " n" ) );
  } else if( C_LOAD < 1E3 ) {
    Serial.print( C_LOAD * 1E6 , 8 );
    Serial.print( F( " u" ) );
  } else if( C_LOAD < 1E0 ) {
    Serial.print( C_LOAD * 1E3 , 8 );
```

```
    Serial.print( F( " m" ) );
  } else {
    Serial.print( C_LOAD , 8 );
    Serial.print( F( " " ) );
  }
  Serial.println( F( "F" ) );

  // Discharge the capacitance
  delay( T * 1000 );
}
```

A.4 – High-level routine to measure a series RC-network in C++ language

```
// Arduino built-in a series RC-network meter according to Section 2.6

#include <SPI.h>

// Assign analog pin A0 to port PA0 at eq. circuit shown in Figure 1(f)
#define PA0  A0
// Define analog pin A3 to port PA1 at eq. circuit shown in Figure 1(f)
#define PA1  A3
// Maximum ADC value considering the 10-bit (n) of resolution: 2^(n)-1
#define NMAX 1023.0
// Analog input resistance value [Ohm(Omega)], see Table 2
#define RAIN 5.451E+06
// Pullup resistance value [Ohm(Omega)], see Table 2
#define RPU  36.66E3
// Output resistance value [Ohm(Omega)], see Table 2
#define ROUT 542.2
// BaudRate of the serial interface. Typical value is 9600, and up to 2E6
#define RxTx 2E6

void setup() {
  // Start serial monitor
  Serial.begin( RxTx );

  // Set-up port PA0 to low output
       pinMode( PA0, OUTPUT );
  digitalWrite( PA0,    LOW );

  // Set-up port PA1 to low output
       pinMode( PA1, OUTPUT );
  digitalWrite( PA1,    LOW );

  // Force the discharge of all capacitances
  delay( 1000 );
}

void loop() {
  // Set-up port PA0 to input pull-up
  pinMode( PA0 , INPUT_PULLUP );

  // Measure the discrete value of the analog port PA1
  float VR = analogRead( PA0 );
```

```
// Read timer in microseconds (us)
unsigned long TS = micros();

// Estimated new reference voltage
float VREF = NMAX - VR;

// Estimated voltage after elapsed one time constant (tau)
float VTAU = VR + 0.6322 * VREF;

// Measure the discrete value of the analog port PA0 until VC < VTAU
float VC = 0;
do {
    VC = analogRead( PA0 );
} while ( VC < VTAU );

// Read timer in microseconds (us)
unsigned long TE = micros();

// Set-up port PA0 to low output
     pinMode( PA0, OUTPUT );
digitalWrite( PA0,    LOW );

// Calculate elapsed time, Delta_t in seconds (s)
float T = ( (float)( TE / 1000L ) - (float)( TS / 1000L ) ) / 1000;

// Calculate variable K using Eq.(2)
float K = RPU * VR / ( NMAX - VR );

// Calculate load resistance (R_LOAD) using Eq.(2)
float R_LOAD = ( ( RAIN + ROUT ) * K - RAIN * ROUT ) / ( RAIN - K );

// Calculate total resistance (RT) value
float RT = R_LOAD + ROUT + ( RAIN * RPU / ( RAIN + RPU ) );

// Calculate load capacitance (C_LOAD) using Eq.(10)
float C_LOAD = T / ( RT * log( VREF / ( VREF - VC ) ) );

// Print resistance value to the serial interface
Serial.print( F( "Resistance: " ) );
if( R_LOAD >= 1E6 ) {
  Serial.print( R_LOAD * 1E-6 , 8 );
  Serial.print( F( " M" ) );
} else if( R_LOAD >= 1E3 ) {
  Serial.print( R_LOAD * 1E-3 , 8 );
  Serial.print( F( " k" ) );
} else {
  Serial.print( R_LOAD , 8 );
  Serial.print( F( " " ) );
}
Serial.print( F( "Ohm   " ) );

// Print capacitance value to the serial interface
Serial.print( F( "Capacitance: " ) );
if( C_LOAD < 1E-12 ) {
  Serial.print( C_LOAD * 1E15 , 8 );
  Serial.print( F( " f" ) );
} else if( C_LOAD < 1E9 ) {
  Serial.print( C_LOAD * 1E12 , 8 );
  Serial.print( F( " p" ) );
} else if( C_LOAD < 1E6 ) {
```

```
    Serial.print( C_LOAD * 1E9 , 8 );
    Serial.print( F( " n" ) );
  } else if( C_LOAD < 1E3 ) {
    Serial.print( C_LOAD * 1E6 , 8 );
    Serial.print( F( " u" ) );
  } else if( C_LOAD < 1E0 ) {
    Serial.print( C_LOAD * 1E3 , 8 );
    Serial.print( F( " m" ) );
  } else {
    Serial.print( C_LOAD , 8 );
    Serial.print( F( " " ) );
  }
  Serial.println( F( "F" ) );

  // Discharge the capacitance
  delay( T * 1E3 );
}
```

A.5 – High-level routine to measure a parallel RC-network in C++ language

```
// Arduino built-in a parallel RC-network meter according to Section 2.7

#include <SPI.h>

// Assign analog pin A0 to port PA0 at eq. circuit shown in Figure 1(f)
#define PA0  A0
// Define analog pin A3 to port PA1 at eq. circuit shown in Figure 1(f)
#define PA1  A3
// Maximum ADC value considering the 10-bit (n) of resolution: 2^(n)-1
#define NMAX 1023.0
// Analog input resistance value [Ohm(Omega)], see Table 2
#define RAIN 5.451E+06
// Pullup resistance value [Ohm(Omega)], see Table 2
#define RPU  36.66E3
// Output resistance value [Ohm(Omega)], see Table 2
#define ROUT 542.2
// BaudRate of the serial interface. Typical value is 9600, and up to 2E6
#define RxTx 2E6

void setup() {
  // Start serial monitor
  Serial.begin( RxTx );

  // Set-up port PA0 to low output
       pinMode( PA0, OUTPUT );
  digitalWrite( PA0,    LOW );

  // Set-up port PA1 to low output
       pinMode( PA1, OUTPUT );
  digitalWrite( PA1,    LOW );

  // Force the discharge of all capacitances
  delay( 1000 );
}
```

```
void loop() {
  // Estimated load resistance (R_LOAD) and capacitance (C_LOAD) values
  float R_LOAD = 1E1;
  float C_LOAD = 1E-6;

  // Estimated total resistance (RT) value
  float RT = RAIN * RPU / ( RAIN + RPU );
        RT = RT * ( ROUT + R_LOAD) / ( RT + ( ROUT + R_LOAD ) );

  // Estimated time constant (tau) of the RC circuit in milliseconds (ms)
  float tau = RT * C_LOAD * 1000;

  // Set-up port PA0 to input pull-up
  pinMode( PA0 , INPUT_PULLUP );

  // Read timer in microseconds (us)
  unsigned long TS = micros();

  // Wait until t > TAU
  delay( tau );

  // Read timer in microseconds (us)
  unsigned long TE = micros();

  // Measure the discrete value of the analog port PA0
  float VC = analogRead( PA0 );

  // Measure elapsed time, Delta_t in seconds (s)
  float T = ( (float)( TE / 1000L ) - (float)( TS / 1000L ) ) / 1000;

  // Wait until t > 5 * TAU, NOTE: it already elapsed t = tau
  delay( tau * 4 );

  // Measure the discrete value of the analog port PA0
  float VF = analogRead( PA0 );

  // Set-up port PA0 to low output
       pinMode( PA0, OUTPUT );
  digitalWrite( PA0,    LOW );

  // Calculate variable K using Eq.(2)
  float K = ( RPU * VF ) / ( NMAX - VF );

  // Calculate load resistance (R_LOAD) using Eq.(2)
  R_LOAD = ( ( RAIN + ROUT ) * K - RAIN * ROUT ) / ( RAIN - K );

  // Calculate total resistance (RT) value
  RT = RAIN * RPU / ( RAIN + RPU );
  RT = RT * ( ROUT + R_LOAD) / ( RT + ( ROUT + R_LOAD ) );

  // Calculate load capacitance (C_LOAD) using Eq.(12)
  C_LOAD = T / ( RT * log( VF / ( VF - VC ) ) );

  // Print resistance value to the serial interface
  Serial.print( F( "Resistance: " ) );
  if( R_LOAD >= 1E6 ) {
    Serial.print( R_LOAD * 1E-6 , 8 );
    Serial.print( F( " M" ) );
  } else if( R_LOAD >= 1E3 ) {
    Serial.print( R_LOAD * 1E-3 , 8 );
    Serial.print( F( " k" ) );
```

```
  } else {
    Serial.print( R_LOAD , 8 );
    Serial.print( F( " " ) );
  }
  Serial.print( F( "Ohm    " ) );

  // Print capacitance value to the serial interface
  Serial.print( F( "Capacitance: " ) );
  if( C_LOAD < 1E-12 ) {
    Serial.print( C_LOAD * 1E15 , 8 );
    Serial.print( F( " f" ) );
  } else if( C_LOAD < 1E9 ) {
    Serial.print( C_LOAD * 1E12 , 8 );
    Serial.print( F( " p" ) );
  } else if( C_LOAD < 1E6 ) {
    Serial.print( C_LOAD * 1E9 , 8 );
    Serial.print( F( " n" ) );
  } else if( C_LOAD < 1E3 ) {
    Serial.print( C_LOAD * 1E6 , 8 );
    Serial.print( F( " u" ) );
  } else if( C_LOAD < 1E0 ) {
    Serial.print( C_LOAD * 1E3 , 8 );
    Serial.print( F( " m" ) );
  } else {
    Serial.print( C_LOAD , 8 );
    Serial.print( F( " " ) );
  }
  Serial.println( F( "F" ) );

  // Discharge the capacitance
  delay( 5 * T * 1E3 );
}
```

A.6 – High-level routine to measure an isolated inductance in C++ language

```
// Arduino built-in an isolated inductance meter through the transient
// acquisition mode, and according to Section 2.8

#include <SPI.h>

// Assign analog pin A0 to port PA0 at eq. circuit shown in Figure 1(f)
#define PA0  A0
// Define analog pin A3 to port PA1 at eq. circuit shown in Figure 1(f)
#define PA1  A3
// Maximum ADC value considering the 10-bit (n) of resolution: 2^(n)-1
#define NMAX 1023.0
// Analog input resistance value [Ohm(Omega)], see Table 2
#define RAIN 5.451E+06
// Pullup resistance value [Ohm(Omega)], see Table 2
#define RPU  36.66E3
// Output resistance value [Ohm(Omega)], see Table 2
#define ROUT 542.2
// BaudRate of the serial interface. Typical value is 9600, and up to 2E6
#define RxTx 2E6

// Inductance value
float L_LOAD      = 0;
```

```
// Measured discrete value by the ADC unit
float NA1          = 0;
// Measured elapsed time until TTL unit return logic '0'
float T            = 0;
// Reference time, supporting variables
unsigned long TS   = 0;
unsigned long TE   = 0;

void setup() {
  // Start serial monitor
  Serial.begin( RxTx );

  // Set-up port PA0 to low output
       pinMode( PA0, OUTPUT );
  digitalWrite( PA0,    LOW );

  // Set-up port PA1 to low output
       pinMode( PA1, OUTPUT );
  digitalWrite( PA1,    LOW );

  // Force the discharge of all inductances
  delay( 1000 );
}

void loop() {
  // Set-up port PA1 to input pull-up
  pinMode( PA1 , INPUT_PULLUP );

  // Read timer in microseconds (us)
  TS = micros();

  // Wait until TTL unit return logic '0'
  do {
      NA0 = digitalRead(PA0);
  } while (NA0 > 0.0);

  // Read timer in microseconds (us)
  TE = micros();

  // Measure the discrete value of the analog port PA0
  NA0 = analogRead( PA0 );

  // Set-up port PA0 to low output
       pinMode( PA0, OUTPUT );
  digitalWrite( PA0,    LOW );

  // Calulate elapsed time, Delta_t in seconds (s)
  float T = ( (float)( TE / 1000L ) - (float)( TS / 1000L ) ) / 1000;

  // Calculate total resistance (RT) value
  float RT = ROUT + ( RAIN * RPU ) / ( RAIN + RPU );

  // Calculate load inductance (L_LOAD) using Eq.(14)
  L_LOAD = T * RT /  log( NMAX / ( NMAX - NA0 ) );
  // Print inductance value to the serial interface
  Serial.print( F( "Inductance: " ) );
  if( L_LOAD < 1E-12 ) {
    Serial.print( L_LOAD * 1E15 , 8 );
    Serial.print( F( " f" ) );
  } else if( L_LOAD < 1E9 ) {
    Serial.print( L_LOAD * 1E12 , 8 );
```

```
    Serial.print( F( " p" ) );
  } else if( L_LOAD < 1E6 ) {
    Serial.print( L_LOAD * 1E9 , 8 );
    Serial.print( F( " n" ) );
  } else if( L_LOAD < 1E3 ) {
    Serial.print( L_LOAD * 1E6 , 8 );
    Serial.print( F( " u" ) );
  } else if( L_LOAD < 1E0 ) {
    Serial.print( L_LOAD * 1E3 , 8 );
    Serial.print( F( " m" ) );
  } else {
    Serial.print( L_LOAD , 8 );
    Serial.print( F( " " ) );
  }
  Serial.println( F( "H" ) );

  // Discharge the inductance
  digitalWrite( PA0,   HIGH );
  delay( T * 1E3 );
  digitalWrite( PA0,   LOW );
}
```

B.1 – High-level routine for fitting the Ordinary Least Squares (OLS) function to the resistance values in MATLAB language

```
function [RAIN,RPU,ROUT] = fittingOLS2R( NMAX , NA0 , RNOMINAL , guessR )
    % Default values according to datasheet of: RAIN, RPU and ROUT
    % Consult Table 1
    RAIN = 100E6;
    RPU  = 39E3;
    ROUT = 600;
    if nargin == 3
        guessR = [ RPU RAIN ROUT ];
    end % END if( nargin )
    % Define the fit function (func) through three parameters to fit.
    % It is easier to gather them in vector R = [R(1) R(2) R(3)]
    % R(1) = RPU
    % R(2) = ROUT
    % R(3) = RAIN

    % The output of func is the estimate of R_LOAD from the PA0 reading
    func = @(R,A0) ( R(1) .* A0 ./ ( NMAX - A0 ) .* ( R(2) + R(3) ) -
                  R(2) * R(3) ) ./ ( R(3) - R(1) .* A0 ./ ( NMAX - A0 ) );

    % Our "x" is the measured NA0 input values
    A0 = NA0;

    % Our "y", is the nominal values of the resistors
    y = RNOMINAL;

    % Define OLS function
    OLS = @(R) sum( ( log( func( R , A0 ) ) - log( y ) ) .^ 2 );

    % Options for fminsearch
    opts = optimset( 'MaxFunEvals' , 600000 , 'MaxIter' , 100000 );

    % Apply fminsearch to find the minimum of the cost function using the
    % guessR input
```

```
    [ RFIT , err ] = fminsearch( OLS , guessR , opts ) ;

    % Use 'fminsearch' to minimise the 'OLS' function
    RPU  = RFIT(1);
    ROUT = RFIT(2);
    RAIN = RFIT(3);

end % END fittingOLS2R()
```

B.2 – High-level routine for fitting the Ordinary Least Squares (OLS) function to the capacitance values in MATLAB language

```
function CPIN = fittingOLS2C( NMAX , NA0 , RNOMINAL , guessC )
    % Default CPIN value according to datasheet, consult Table 1
    CPIN = 24.48E-12;
    if nargin == 3
        guessC = CPIN;
    end % END if( nargin )
    % Define the fit function (func) through the parameters to fit:
    % C(1) = CPIN


    % The output of func is the estimate of C_LOAD from the PA0 reading
    func = @(C,A0) C(1) .* A0 ./ ( NMAX - A0 );

    % Our "x" is the measured NA0 input values
    A0 = NA0;

    % Our "y", is the nominal values of the capacitors
    y = CNOMINAL;

    % Define OLS function
    OLS = @(C) sum( ( log( func( C , A0 ) ) - log( y ) ) .^ 2 );

    % Options for fminsearch
    opts = optimset( 'MaxFunEvals' , 600000 , 'MaxIter' , 100000 );

    % Apply fminsearch to find the minimum of the cost function using the
    % guessC input
    [ CFIT , err ] = fminsearch( OLS , guessC , opts );

    % Use 'fminsearch' to minimise the 'OLS' function
    CPIN  = CFIT(1);
end % END fittingOLS2C()
```
